# Metabolic Regulation of Dimethylsulfoniopropionate Cleavage and Dimethyl Sulfide Production in *Halomonas* sp. D47

**DOI:** 10.1002/advs.202514858

**Published:** 2026-02-13

**Authors:** Li‐Yuan Zheng, Wen‐Xin Jiang, Xiao‐Meng Sun, Li Liu, Zhao‐Jie Teng, Hou‐Qi Wang, Wen‐Jing Zhu, Chang Ge, Qi‐Long Qin, Ning‐Hua Liu, Hai‐Yan Cao, Hui‐Hui Fu, Chun‐Yang Li, Jonathan D. Todd, Xiu‐Lan Chen, Yu‐Zhong Zhang, Peng Wang

**Affiliations:** ^1^ Marine Biotechnology Research Center State Key Laboratory of Microbial Technology Shandong University Qingdao China; ^2^ MOE Key Laboratory of Evolution and Marine Biodiversity Frontiers Science Center For Deep Ocean Multispheres and Earth System & College of Marine Life Sciences Ocean University of China Qingdao China; ^3^ School of Rehabilitation Sciences and Engineering University of Health and Rehabilitation Sciences Qingdao China; ^4^ Laboratory For Marine Biology and Biotechnology Qingdao Marine Science and Technology Center & Laoshan Laboratory Qingdao China; ^5^ School of Biological Sciences University of East Anglia Norwich UK

**Keywords:** anti‐greenhouse gas, dimethyl sulfide, dimethylsulfoniopropionate, sulfur cycle, transcriptional regulation

## Abstract

Dimethylsulfoniopropionate (DMSP) is a globally significant marine organosulfur compound with diverse ecological roles, including environmental stress protection, chemotaxis, and nutrient cycling. Its microbial catabolism is crucial for the marine sulfur cycle, generating dimethyl sulfide (DMS), a volatile gas that influences global sulfur fluxes, cloud formation, and climate regulation. Despite its importance, the metabolic regulatory mechanisms governing bacterial DMSP cleavage and DMS production remain unclear. Here, using the model DMSP‐catabolizing bacterium *Halomonas* sp. D47, a complex regulatory mechanism involving two transcriptional regulators, AcuR and AcuZ, was elucidated through integrated genetic and biochemical analyses, in which they coordinate the orderly progression of DMSP catabolism. These regulators sense external signals from DMSP and its metabolites, fine‐tuning gene expression to balance metabolism and detoxification, thereby maintaining cellular integrity. Bioinformatics analyses suggest that this regulatory scheme is conserved among certain efficient DMSP‐metabolizing bacteria. Our findings provide key insights into the regulation of DMSP catabolism and highlight a potentially bacterial strategy for balancing metabolic demands with cellular homeostasis.

## Graphical abstract

Dimethylsulfoniopropionate (DMSP) is a major marine organosulfur compound central to climate‐relevant dimethyl sulfide (DMS) production. In *Halomonas* sp. D47, DMSP catabolism is revealed to be coordinated by two transcriptional regulators, AcuR and AcuZ, which control gene expression by sensing DMSP and its metabolites. This regulatory strategy, conserved in some efficient degraders, balances metabolism, detoxification, and cellular homeostasis.

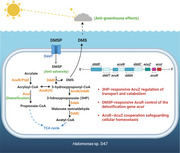

## 1 Introduction

Billions of tonnes of the zwitterion dimethylsulfoniopropionate (DMSP) are produced annually by many marine phytoplankton, bacteria, and coastal angiosperms [1, 2, 3, 4, 5]. DMSP can represent up to 10% of the carbon that is fixed by phytoplankton cells [1]. These organisms produce DMSP as an antistress molecule to protect against salinity, hydrostatic pressure, and oxidative stress [2, 6]. When released into the environment, DMSP also plays important roles in ecological and metabolic interactions between phytoplankton and heterotrophic bacteria, and serves as a key source of carbon and sulfur via two distinct DMSP catabolic pathways [7, 8]. Among these, the cleavage pathway generates over 300 Tg of dimethyl sulfide (DMS) annually, along with a three‐carbon co‐product—either acrylate or 3‐hydroxypropionate (3‐HP), depending on the specific DMSP lyase involved [1, 2]. These cleavage products can be important signaling molecules to attract or repel specific grazers, influencing marine prey–predator dynamics [9]. Furthermore, the produced DMS accounts for approximately 90% of total marine sulfur emissions and is a climate‐active gas whose oxidation products can also act as cloud condensation nuclei (CCN), influencing the Earth's solar radiation budget and are transferred back to land in rain [10, 11, 12, 13, 14]. Furthermore, the coupling of DMS and halogen ocean emissions can significantly impact the air quality of coastal megacities [15].

To date, there is one known algal DMSP lyase, termed Alma [16]. In contrast, no fewer than eight different enzymes, termed DddL, DddP, DddQ, DddW, DddK, DddY, DddX, and DddU, have been found in diverse bacteria—particularly genus *Alcaligenes*, *Pseudomonas*, *Ruegeria, Candidatus *Pelagibacter, *and Sulfitobacter* in Proteobacteria to cleave DMSP into DMS plus acrylate or acryloyl‐CoA [17, 18, 19, 20, 21, 22, 23]. One additional bacterial lyase, DddD, which is described below, produces 3‐HP or 3‐HP‐CoA as its C‐3 co‐product, rather than acrylate or acryloyl‐CoA [24, 25, 26].

Several lines of evidence point to the importance of the DddD lyase. The bacteria that contain this gene, which are mostly restricted to marine γ‐Proteobacteria, grow very well on DMSP as a sole carbon source. Also, *dddD* is usually located in a cluster of other genes that encode other relevant functions, involved in the uptake of DMSP, in downstream catabolic steps, and in the DMSP‐dependent transcriptional regulation of the *ddd* genes in the cluster. In contrast, most other DMSP lyases are not associated with DMSP catabolic gene clusters, and they are not always tightly regulated by DMSP or its catabolites [19, 23]. Furthermore, the DddD lyase was shown to have significantly higher enzyme activity than most of the other lyases [20]. And finally, a stable isotope probing (SIP) study of a natural sample from coastal waters at Great Yarmouth, UK, identified members of the order Oceanospirillales within the class γ‐proteobacteria, including the genera *Amphritea*, *Marinomonas*, *Marinobacterium*, and *Oceanospirillum*, as the dominant microorganisms utilizing DMSP as a carbon source, with the DddD lyase being the primary enzyme involved [27].

DddD was the first of the DMSP lyases to be discovered, having been found in the bacterium *Marinomonas* MWYL1 [24]. Another member of the Oceanospirillales, namely *Halomonas* sp. HTNK1 was also found to grow well on DMSP thanks to its DddD lyase [27]. However, in contrast to *Marinomonas*, *Halomonas* HTNK1 also grew well on acrylate as the sole carbon source, even though this strain did not generate this C‐3 molecule from DMSP. The ability to catabolize acrylate is mediated by the products of two other genes, *acuN* and *acuK*, which are closely linked to the *ddd* genes and which encode an acryloyl CoA transferase (AcuN) and a hydratase (AcuK) that act together to generate 3‐HP [28].

Todd et al. 2010 used *lac* fusions to show that the expression of the *acu* and *ddd* genes of *Halomonas* HTNK1 was raised by a factor of at least 30‐fold, not only by the substrates DMSP and acrylate, but also by the catabolic product 3‐HP. Two potential regulatory genes, termed *dddZ* and *acuR*, were identified in the gene cluster, the sequences of their products placing them respectively in the LysR and TetR families of transcriptional regulators [28]. In *Marinomonas* MWYL1, the expression of its *ddd* genes was also greatly enhanced in the presence of DMSP, this being mediated by the transcriptional activator DddR [24]. DddR is also in the LysR super‐family, but its sequence is not particularly similar (47.5% identity) to that of the *Halomonas* HTNK1 DddZ polypeptide [24]. Until now, although potential transcriptional regulators have been identified, the direct effectors and detailed metabolic regulatory mechanisms remain unclear.

*Halomonas* sp. D47, a γ‐Proteobacterium capable of catabolizing DMSP, was isolated by our group from a sediment sample collected at Taipingjiao, Qingdao, China, using DMSP as the sole carbon source. In this study, genome sequencing and bioinformatic analysis allowed us to identify the putative DMSP‐catabolizing gene cluster and its associated regulatory proteins, AcuR and AcuZ. The functions of AcuR and AcuZ were subsequently characterized through molecular biology and biochemical experiments, including heterologous expression and functional assays. Based on these combined approaches, we propose a collaborative mechanism by which AcuR and AcuZ coordinate DddD‐mediated DMSP catabolism and DMS production in strain D47, and our analyses suggest that this regulatory mechanism is likely conserved among certain efficient DMSP‐catabolizing bacteria.

## 2 Results and Discussion

### 2.1 The DMSP Catabolism Gene Cluster in *Halomonas sp*. D47

The 16S rRNA gene sequence of strain D47 was found to be identical to that of *Halomonas* sp. HTNK1 (100% identity; 100% coverage for the alignment) [28]. Similar to *Halomonas* sp. HTNK1, strain D47, could grow on media containing 5 mM DMSP or acrylate as the sole carbon source and, indeed, grew better on the acrylate. At 24 h (stationary phase), the OD_600_ on acrylate as the sole carbon source was 3.01‐fold higher than on DMSP (Figure S1a).

**FIGURE 1:**
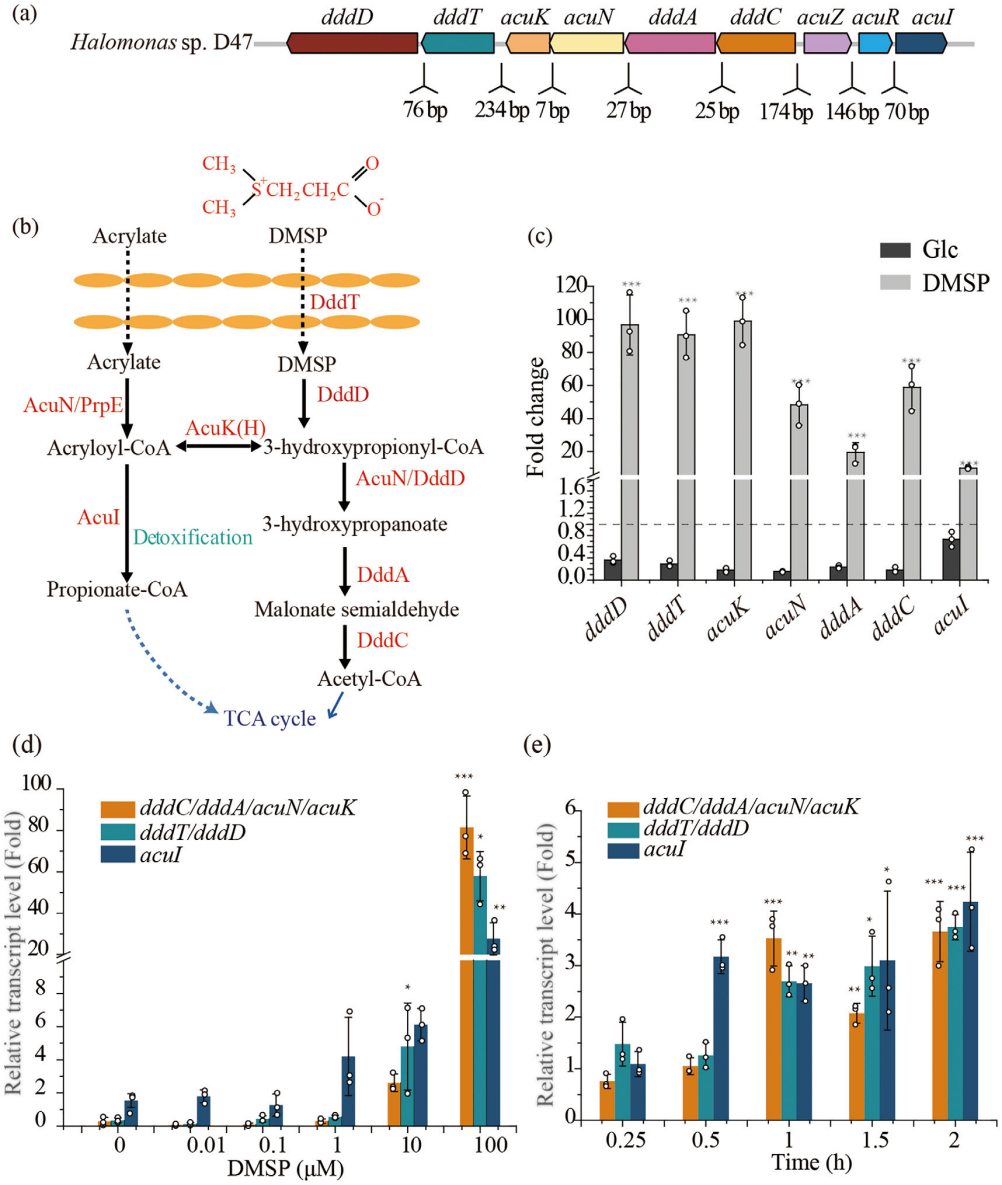
DMSP utilization in strain D47 and the associated transcriptional response. (a) DMSP/acrylate catabolic gene cluster in strain D47. (b) Proposed DMSP catabolic pathway in strain D47, highlighting key enzymes in red. (c) Transcriptomic analysis of the gene expression levels in strain D47 when grown on 5 mM DMSP versus glucose (Glc) as the sole carbon source, compared with 0 h sample. Samples were collected at 0 h and when the cells had reached the mid‐logarithmic growth phase. (d) Changes in transcriptional levels of DMSP degradation and transport genes in strain D47 upon exposure to varying DMSP concentrations. Samples were collected at 0 h and when the cells had reached the mid‐logarithmic growth phase. (e) Transcriptional profiling of DMSP metabolic and transport genes across different growth phases of strain D47 cultured with 5 mM DMSP as the sole carbon source. Data are presented as mean ± SD from three independent experiments. To assess statistical differences between groups, a two‐tailed *t*‐test was applied. *p*‐values in panels (c), (d), and (e) were calculated by comparison with the Glc group, the 0 µM DMSP group, and the 0.25 h group, respectively. * *p* < 0.05; ** *p* < 0.01; *** *p* < 0.001 indicate levels of significance; ns denotes a nonsignificant difference (*p* ≥ 0.05). *p*‐values were referenced against the corresponding Glc group (c) or the 0 h samples (d, e).

The genome of strain D47 was sequenced, revealing a DMSP catabolic cluster that includes *dddD*, *dddT*, *acuK*, *acuN*, *dddA*, *dddC*, *acuZ*, *acuR*, and *acuI*, which is identical to the cluster found in *Halomonas* sp. HTNK1 (*dddZ* in *Halomonas* sp. HTNK1 has now been renamed *acuZ*) (Figure 1a,b; Table S1). Among these genes, *dddD* encodes the DMSP lyase DddD, while *dddT* encodes the DMSP transporter *DddT*. *acuK* and *acuN* are involved in acrylate metabolism, whereas *dddA* and *dddC* contribute to downstream metabolic processes. *acuI* encodes an acryloyl‐CoA reductase, which has been proposed in other bacteria to detoxify the highly toxic acryloyl‐CoA formed during DMSP and/or acrylate catabolism [29, 30] (Figure 1b). *acuZ* and *acuR* encode transcriptional regulators, suggesting their roles in the regulation of DMSP catabolism.

### 2.2 The Response of the DMSP Catabolic Gene Cluster to DMSP and Its Metabolites

Examination of the sequence of the *ddd/acu* gene cluster revealed four significant intergenic gaps upstream of *dddT*, *dddC*, *acuR*, and *acuI* (Figure 1a; Figure S1b). Furthermore, the PROM program (http://linux1.softberry.com) predicted promoters upstream of each of these, but not in the region upstream of *dddD*, *dddA*, *acuN*, and *acuK* (Figure 1a; Figure S1b). To confirm that each of the gaps indeed contains at least one transcriptional start site, PCR was carried out on adjacent genes using cDNA template and primer pairs that spanned various intergenic regions (Figure S1c). DNA products were only obtained when the intervening DNA between the two primers spanned the gap regions described above. From this, we infer that *dddT‐dddD* are co‐transcribed, as are *dddC‐dddA‐acuN‐acuK*, whereas *acuZ*, *acuR*, and *acuI* are each in mono‐cistronic units.

To investigate how DMSP and its metabolic intermediates influence the expression of genes involved in their transport and catabolism, *Halomonas* sp. D47 cells were subjected to transcriptomic analyses. Strain D47 cells were incubated at 4°C for 2 h and subsequently inoculated separately into minimal medium containing DMSP or glucose (Glc). Samples were collected at *t* = 0 h and at the midpoint of the logarithmic growth phase for transcriptome analysis. The results showed that when DMSP was the sole carbon source, except for regulator genes *acuZ* and *acuR*, transcripts from the six catabolic (*dddA, C, D*, and *acuN, K and I*) and transport (*dddT*) genes in the midpoint growth sample were much (>fourfold) more abundant compared to the 0‐hour sample (Figure 1c). Furthermore, except for *acuI*, transcription of these *ddd* and *acu* genes was also significantly downregulated when cultured with Glc compared to the 0‐h sample (Figure 1c). These findings were further validated through RT‐qPCR analysis (Figure S1d). It was also found that the transcriptional levels of these genes were up‐regulated when cultured with two of the metabolic intermediates, acrylate and 3‐HP, which were also examined by RT‐qPCR (Figure S1e). Similar to the results reported by Todd et al. 2010, these findings confirmed that genes for the transport and catabolism of DMSP were induced by DMSP and/or its catabolites, although the specific effectors need to be further detected.

The above experiments were conducted with mM levels of DMSP, which exist in many DMSP producers and consumers through DMSP import and accumulation [31, 32], but DMSP concentrations in epipelagic seawaters vary from nanomolar to rarer micromolar levels [33]. Thus, we also examined the response of strain D47 to lower, more physiologically relevant DMSP levels. Growth of strain D47 was only noticeable when the DMSP exceeded 1 mM (sole carbon source) (Figure S1f), whereas 1 µM DMSP levels induced significant up‐regulation of *acuI* transcription (Fold change > 2; *p* value < 0.05). In contrast, *dddTD* and *dddCA‐acuNK* transcription was only upregulated at concentrations >10 µM DMSP (Figure 1d).

In contrast to *dddTD* and *dddCA‐acuNK*, the acryloyl‐CoA reductase product of *acuI* can detoxify the toxicity of acryloyl‐CoA, a metabolic intermediate in DMSP metabolism [34, 35, 36]. Thus, the heightened sensitivity of *acuI* expression to DMSP is likely associated with the earlier and faster clearance of acryloyl‐CoA. Indeed, in growth experiments, we confirmed that *acuI* transcription was upregulated earlier (0.5 h) compared to *dddTD* and *dddCA‐acuNK* (1 h) (Figure 1e).

### 2.3 The Direct Effectors of AcuZ and Their Binding Mechanisms

Different members of the prominent class of LysR‐type transcriptional regulators (LTTRs) are known to modulate gene expression through effector‐mediated or direct binding to target genes [37, 38, 39]. Sequence analysis has placed AcuZ within the LysR family, albeit with relatively low sequence identity (27%–36%) compared to well‐studied members (Table S2).

**FIGURE 2:**
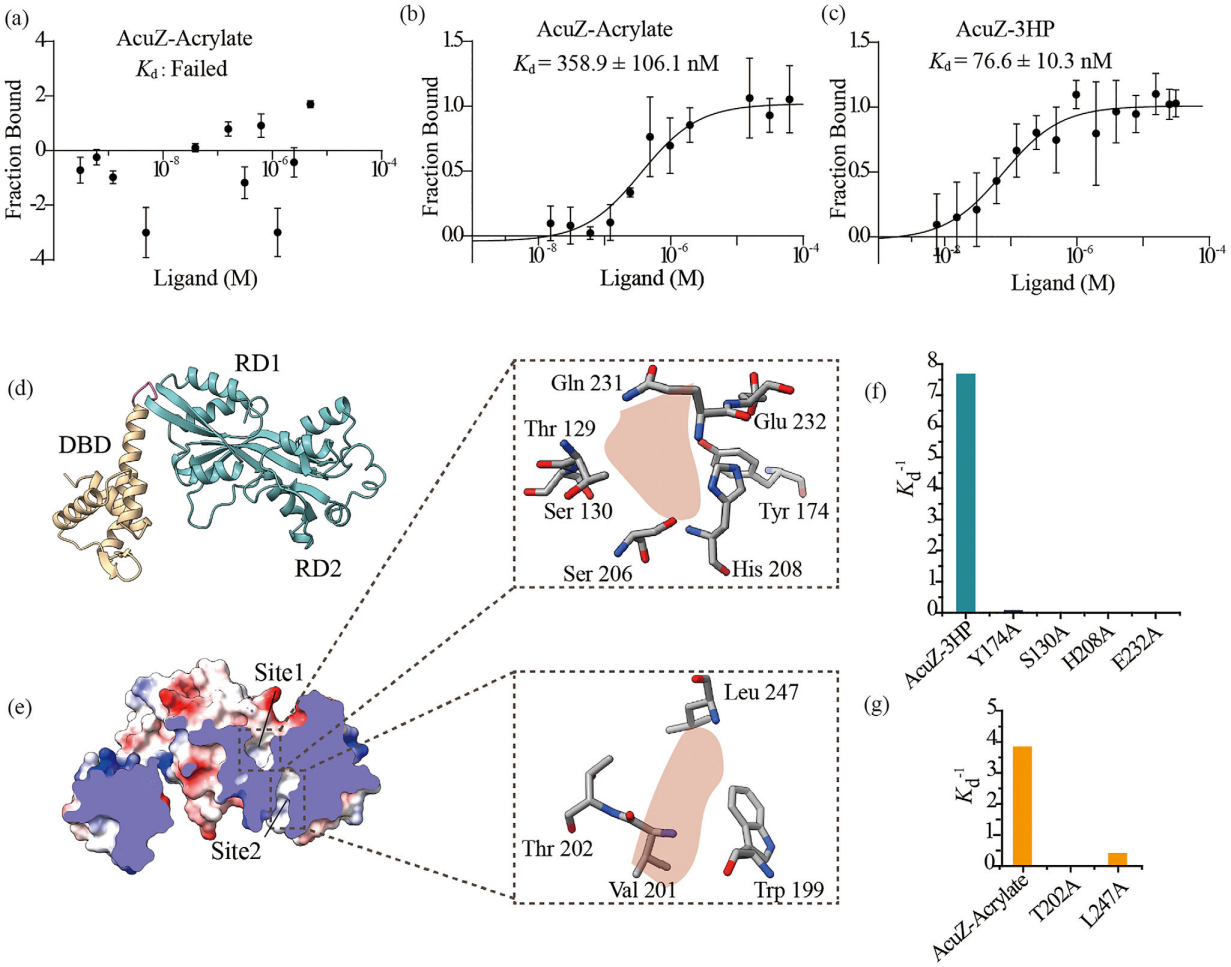
Direct effectors of AcuZ and their binding mechanisms. (a, b, c) Assessment of AcuZ's binding affinity with DMSP (a), acrylate (b), and 3‐HP (c). Data are presented as mean ± SD from three independent experiments. (d) Predicted 3D structure of AcuZ. (e) Identification of putative effector binding sites on AcuZ. Site 1 has specificity for 3‐HP, while site 2 targets acrylate. (f, g) Binding affinity of AcuZ and its mutants with 3‐HP (f) or acrylate (g). The *K*_d_^−1^ was plotted as the ordinate to facilitate a more intuitive representation of the change in binding affinity. The binding affinity with 3‐HP and acrylate was marked with blue and orange, respectively.

It is known that DMSP, acrylate, and 3‐HP all may act as co‐inducers of the *ddd* and *acu* genes in *Halomonas* [26, 28]. We therefore examined the binding affinities of recombinant AcuZ protein to these three molecules using microscale thermophoresis (MST). The results showed that AcuZ can interact with acrylate and with 3‐HP with dissociation constants (*K*_d_) of 358.9 ± 106.1 nM and 76.6 ± 10.3 nM, respectively. However, no binding of AcuZ to DMSP was detected (Figure 2a–c). These results indicated that acrylate and 3‐HP can probably each act as their direct effectors, while DMSP cannot.

Structural and biochemical analyses were performed to elucidate how AcuZ recognizes and binds its effectors, aided by the predicted 3D structure of AcuZ generated by AlphaFold2 (Figure 2d). Each AcuZ subunit is composed of a DNA‐binding domain (DBD) and a regulatory domain (RD), connected by a short hinge region. The DBD contains a winged helix‐turn‐helix (wHTH) motif with four *α*‐helices and two *β*‐strands, while the RD is subdivided into two *α*/*β* Rossman‐like subdomains, RD1 and RD2. Structural inspection revealed two internal hydrophilic cavities located between RD1 and RD2, which are potential effector‐binding sites based on comparisons with homologous proteins. Site 1 is encircled by residues Thr129, Ser130, Tyr174, Ser206, His208, Gln231, and Glu232; Site 2 is surrounded by residues Trp199, Val201, Thr202, and Leu247 (Figure 2e).

To probe the functional importance of these residues, site‐directed mutagenesis was performed. Circular dichroism analysis confirmed that the overall structures of the mutants were similar to the wild‐type protein (Figure S2). Binding assays using MST with each mutant at 10 µM revealed site‐specific effector recognition. Mutation of Ser130, Tyr174, His208, and Glu232 in Site 1 selectively impaired binding to 3‐HP without affecting acrylate recognition (Figure 2e,f; Figure S3). Conversely, mutation of Tyr202 and Leu247 in Site 2 reduced binding to acrylate but not 3‐HP (Figure 2e,g; Figure S3).

These results demonstrate that 3‐HP and acrylate bind to distinct sites on AcuZ. Site 1 mediates 3‐HP recognition, with Ser130, Tyr174, His208, and Glu232 being critical, whereas Site 2 mediates acrylate recognition, with Tyr202 and Leu247 playing key roles. Collectively, these findings provide a structural explanation for AcuZ's ability to simultaneously recognize and respond to both 3‐HP and acrylate as effector molecules, highlighting the molecular basis of its regulatory function.

### 2.4 The Switching of AcuZ Between Activator and Repressor Functions

To explore AcuZ's role, the DNA‐binding regions of AcuZ on the gene cluster were first investigated. Sequences upstream of *dddC*, *dddT*, *acuZ*, *acuR*, and *acuI* were amplified and assessed for their interactions with AcuZ as ligands (Table S3). It was found that AcuZ bound to the sequences upstream of *dddC* and *dddT*, exhibiting dissociation constants (*K*_d_) of 17.9 ± 10.56 nM and 75.81 ± 9.26 nM, respectively (Figure S4). Due to weak binding forces, DNase I footprinting, which was aimed at mapping the binding sites of AcuZ, yielded inconclusive results. This is a common occurrence in the study of LTTRs [39, 40, 41, 42]. To validate the binding of AcuZ to the sequences upstream of *dddC* and *dddT*, promoter mutants (0.2 µM) were incubated with increasing concentrations of AcuZ (0, 4, and 8 µM) and analyzed by electrophoretic mobility shift assays (EMSAs). The mutants *mdddC* and *mdddT* showed no binding shifts (Figure S5), suggesting the necessity of the AATTGATT and TATA‐N8‐GATA palindromic sequences in the core promoter regions for the recognition and binding by AcuZ.

**FIGURE 3:**
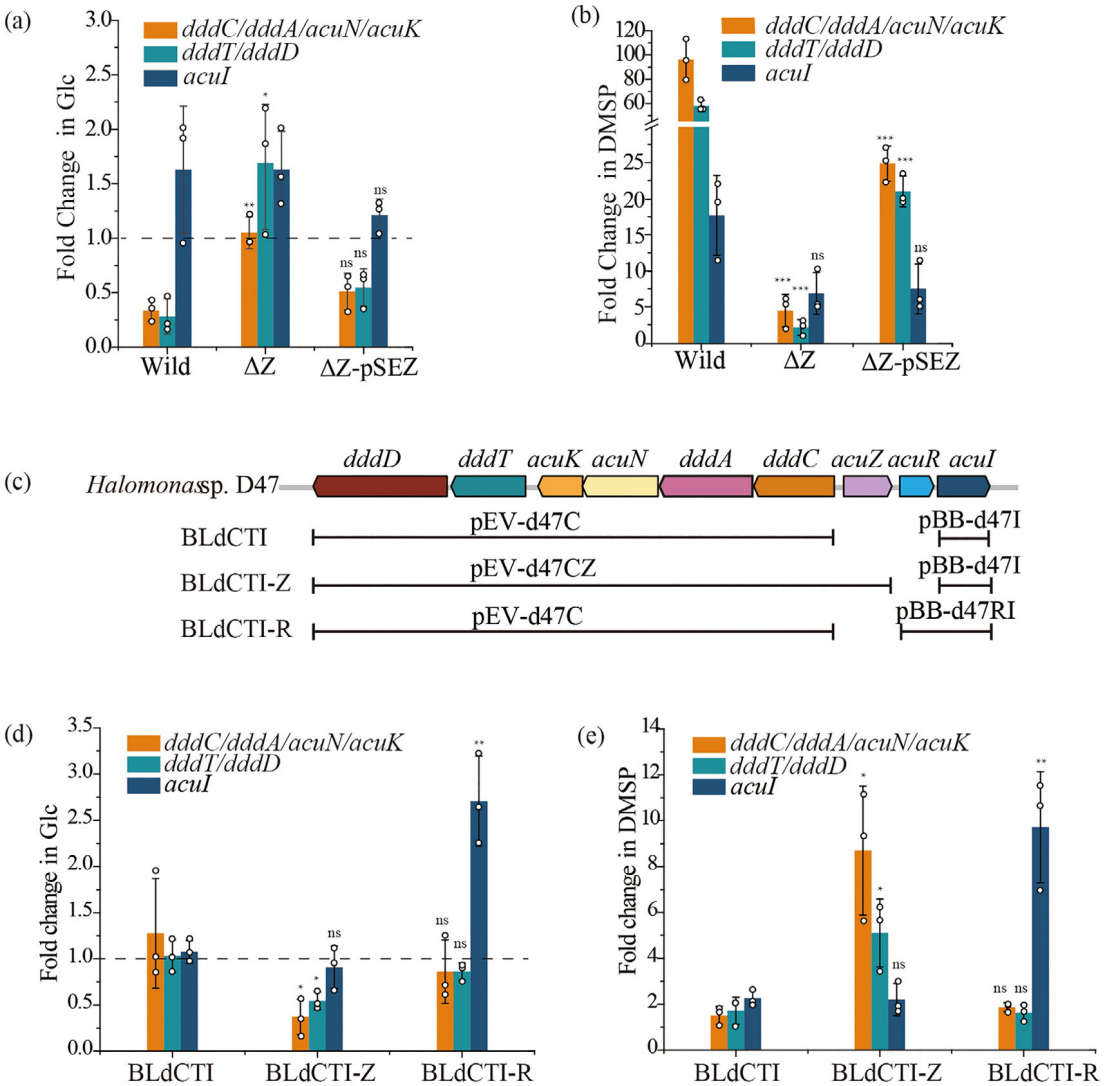
Roles of AcuR and AcuZ in strain D47. (a, b) RT‐qPCR analysis of gene expression levels in strains D47, ΔZ and ΔZ‐pSEZ when grown on 5 mM glucose (a) or DMSP (b) as the sole carbon source compared with 0 h sample. (c) The construction of recombinant *Escherichia coli* strains BLdCTI, BLdCTI‐Z and BLdCTI‐R. (d, e) RT‐qPCR analysis of gene expression in recombinant strains BLdCTI, BLdCTI‐Z and BLdCTI‐R when incubated with glucose (d) or DMSP (e) as the sole carbon source compared with 0 h sample. Samples were collected at 0 h and when the cells had reached the mid‐logarithmic growth phase. Data are presented as mean ± SD from three independent experiments. To determine statistical differences between mutant and wild‐type strains, a two‐tailed *t*‐test was performed. *p*‐values in (a, b) and (d, e) were determined relative to the wild type and BLdCTI, respectively. * *p* < 0.05; ** *p* < 0.01; *** *p* < 0.001 signify levels of significance; ns indicates no significant difference (*P* ≥ 0.05). *p*‐values are referenced to the respective wild type or BLdCTI.

Then, we developed an *acuZ* knockout mutant (ΔZ) and constructed a plasmid, termed pEVZ, in which the *acuZ* gene was cloned in strain D47 (see Materials and methods). The ΔZ mutant's growth on Glc resembled that of the wild type (Figure S6a). Notably, under Glc conditions, ΔZ showed higher transcription of *dddCA‐acuNK* and *dddTD* than the wild‐type strain D47 (Figure 3a), indicating that AcuZ may act as a repressor of *dddTD* and *dddCA‐acuNK* in the absence of DMSP. Conversely, with DMSP as the sole carbon source, ΔZ exhibited slower growth and reduced transcription of these genes compared to the wild type (Figure 3b; Figure S6b). Furthermore, transcription in the complemented ΔZ‐pSEZ strain was partially recovered. This suggests that AcuZ functions as a 3‐HP responsive activator for *dddCD* and *dddCA‐acuNK*.

For further insights into the regulator's role, *Escherichia coli* strain BL21 (DE3) was used as an alternative host for expression studies. We introduced recombinant plasmids into strain BL21 (DE3), in which the cluster's catabolic genes (*dddTD*, *dddCA‐acuNK*, and *acuI*) had been cloned (strain BLdCTI) and which also, in strain BLdCTI‐Z, contained the regulatory *acuZ* gene (Figure 3c). When the cloned *acuZ* gene was present (in BLdCTI‐Z), besides *acuI*, the transcriptional level of *dddTD* and *dddCA‐acuNK* in BLdCTI‐Z was repressed compared with those in BLdCTI in the presence of Glc, while it was induced obviously in the presence of DMSP (Figure 3d,e). These results revealed that AcuZ represses *dddTD* and *dddCA‐acuNK* in glucose environments and activates them in the presence of DMSP.

Thus, AcuZ likely functions as follows: in the absence of effectors (3‐HP/acrylate), AcuZ binds to the upstream regions of the *dddC* and *dddT* promoters, inhibiting the transcription of the catabolic genes *dddTD* and *dddCA‐acuNK*. Upon effector binding, AcuZ undergoes allosteric changes that activate the transcription of the catabolic genes *dddTD* and *dddCA‐acuNK*. This mechanism is similar to the action of the LysR family regulator ClcR in catechol metabolism [41, 42].

### 2.5 The Direct Effectors of AcuR and Their Binding Mechanisms

TetR family transcriptional regulators (TFRs) are ubiquitous in bacteria and archaea, with species harboring larger genomes often encoding a higher number of TFRs [43, 44]. These regulators can serve as local or global modulators of gene expression, functioning as repressors or activators by binding to small molecule or protein effectors [45, 46]. The sequence of AcuR of strain D47 positions it within the TetR family, sharing 24%–54% sequence identity with the detailed studied members (Table S4).

**FIGURE 4:**
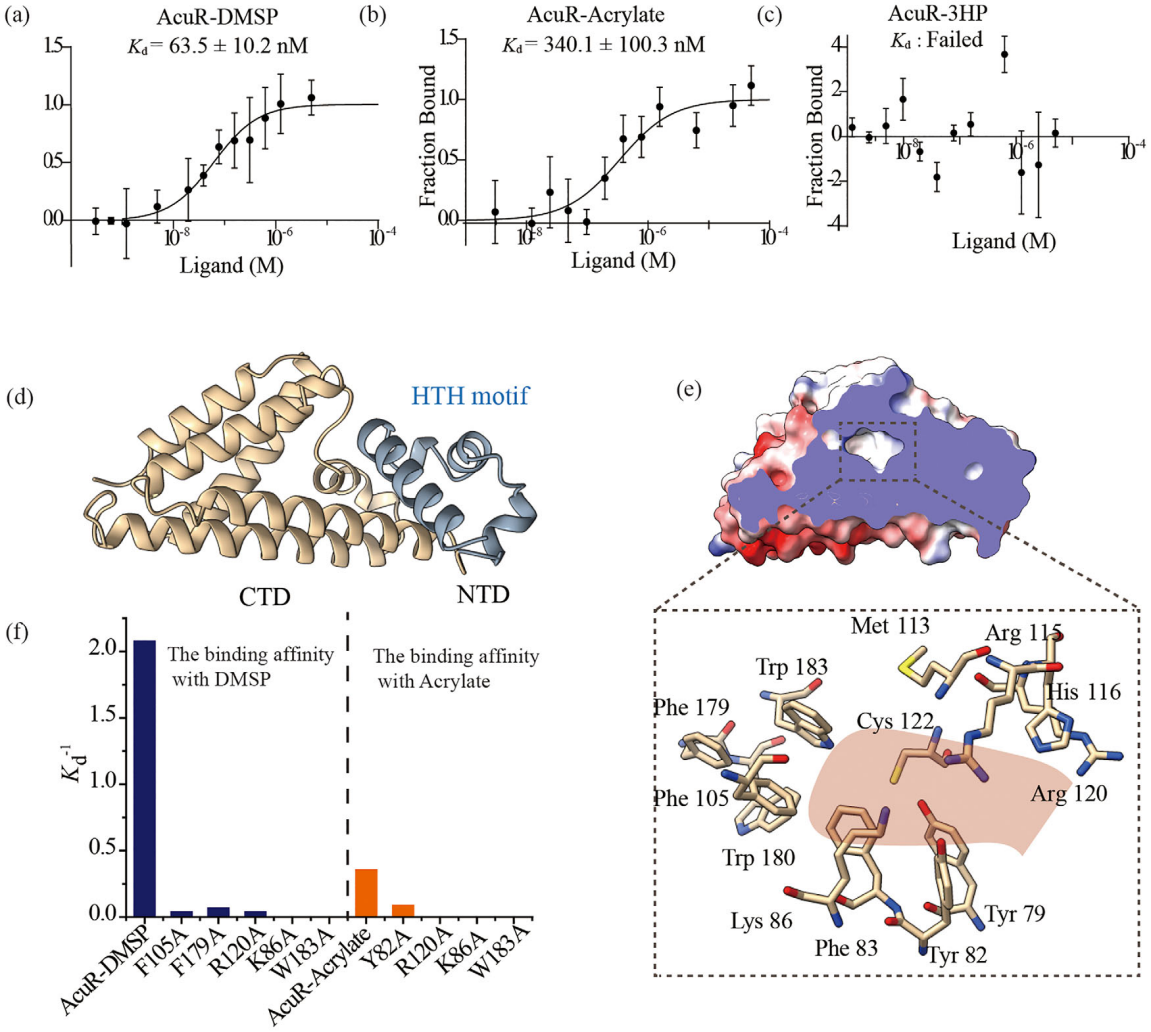
Direct effectors of AcuR and their binding mechanisms. (a, b, c) Examination of AcuR's binding affinity with DMSP (a), acrylate (b), and 3‐HP (c). Data are presented as mean ± SD from three independent experiments. (d) Predicted 3D structure of AcuR. (e) Hypothesized effector binding site on AcuR. (f) Binding affinity of AcuR and its mutants with DMSP or acrylate. The *K*_d_^−1^ was plotted as the ordinate to facilitate a more intuitive representation of the change in binding affinity. The binding affinity with 3‐HP and acrylate was marked with blue and orange, respectively.

As was done with the AcuZ regulator, we also examined biochemically if the AcuR polypeptide interacted with DMSP and/or its metabolic intermediates, 3‐HP, and acrylate. MST results indicated that AcuR interacts with both DMSP and acrylate, with dissociation constants (*K*_d_) of 63.5 ± 10.2 nM and 340.1 ± 100.3 nM, respectively (Figure 4a–c), but no binding of 3‐HP was detected. To elucidate the specific recognition and binding sites of DMSP and acrylate by AcuR, structural and biochemical analyses were performed. The Alphafold2 predicted structure of AcuR was found to comprise nine *α*‐helices split into an N‐terminal domain (NTD) featuring a helix‐turn‐helix (HTH) motif, and a C‐terminal domain (CTD) (Figure 4d). A central pocket, characteristic of TetR family regulators, is formed by the residues Tyr79, Tyr82, Phe83, Lys86, Phe105, Met113, Arg115, His116, Arg120, Cys122, Phe179, Trp180, and Trp183, which is postulated to be the effector binding site (Figure 4e). This was validated through site‐directed mutagenesis. Alanine substitutions at Tyr82, Lys86, Arg120, or Trp183 significantly diminished the binding of AcuR to acrylate. In a similar vein, alanine substitutions at Lys 86, Phe105, Arg120, Phe179, or Trp183 markedly reduced the affinity of AcuR for DMSP (Figure 4e,f; Figure S7). CD spectra indicated that the secondary structures of all these mutants were similar to that of the wild‐type AcuR, suggesting that changes in binding affinity were due to residue substitution rather than structural alterations (Figure S2). Although the residues affecting the binding of DMSP and acrylate are not identical, these findings indicate that DMSP and acrylate share a common binding site on AcuR.

### 2.6 The Regulatory Function of AcuR

To explore AcuR's function, investigations were first conducted to identify the DNA‐binding regions of AcuR within the gene cluster and to assess the impact of effectors on its DNA binding. It was discovered that AcuR binds to sequences upstream of *acuI*, exhibiting a dissociation constant (*K*_d_) of 344.3 ± 99.89 nM, which is reduced to 13.8 ± 10.56–27.78 ± 9.02 nM in the presence of effectors (Figure S8). These results indicated that the core promoter of *acuI* is bound by AcuR, and this interaction is intensified when acrylate or DMSP is present as an effector. The sequence TATAGA‐N7‐TCTATA is a characteristic recognition site for TetR family regulators [47, 48, 49]. To confirm the binding of AcuR to target sequences, EMSAs were performed using increasing concentrations of AcuR (0, 10, and 20 µM) and short oligonucleotide probes (0.2 µM) that encompassed the core promoter regions and a mutant sequence *macuI*, derived from documented sequences (Figure S9). These results showed that the DNA probes corresponding to sequences upstream of *acuI* were shifted by purified AcuR, but the *macuI* probe was not affected (Figure S9).

Then, we attempted to create an *acuR* knockout mutant for further study, but this failed, possibly due to ineffective gRNA targeting the *acuR* gene, or it is lethal. If deletion of *acuR* is lethal, this would indicate that AcuR proteins may have a broader or higher‐level function beyond the regulation of the *ddd* genes. Further studies are needed to elucidate the conditional functions of AcuR. We opted for *Escherichia coli* BL21 (DE3) as a substitute host to conduct expression studies, aiming to advance our understanding of AcuR's role. As mentioned above, the recombinant strains BLdCTI and BLdCTI‐R contain distinct genetic compositions. BLdCTI was engineered with the DMSP catabolic cluster's catabolism and transport genes, excluding *acuR* and *acuZ*; and BLdCTI‐R included these genes plus *acuR* (Figure 3c). The RT‐qPCR results showed that when cultured with Glc, the transcriptional level of *acuI* in BLdCTI‐R was increased compared with that in BLdCTI, and was induced obviously in the presence of DMSP (Figure 3d,e). The results suggested that AcuR consistently acts as an activator for *acuI* irrespective of whether glucose or DMSP served as the sole carbon source.

It has been reported that AcuR homologs in the strains *Rhodobacter sphaeroides* 2.4.1 and *Alcaligenes faecalis* M3A act as repressors in the DMSP‐related operons *acuR‐acuI‐dddL* and *acuR‐dddC‐acuZ‐acuI‐dddY*, respectively, rather than as an activator as seen in strain D47 [50, 51]. *R. sphaeroides* 2.4.1 and *A. faecalis* M3A belong to the *α*‐ and *β*‐proteobacteria classes, respectively. The difference in the metabolic operons is that AcuR in strain D47 regulates only the detoxification gene *acuI*, but in *R. sphaeroides* 2.4.1 and *A. faecalis* M3A, AcuR controls both the DMSP lyase (respectively *dddL* and *dddY*) genes as well as *acuI*. The variation in AcuR function among different strains may likely be attributed to the different metabolic and physiological homeostasis requirements of the strains, while the reason needs to be further studied. As a member of the TetR family, AcuR indeed has the potential to perform different functions in different operons. Indeed, despite sequence similarities, the function of each regulator listed in Table S4 was observed to vary with the function of each metabolism. Some act as repressors, some as activators, and in certain cases, dual‐function regulators are also present [52, 53, 54].

Overall, AcuR likely functions as follows: In the absence of effectors (DMSP/acrylate), AcuR is still able to bind the *acuI* promoter, albeit with lower affinity, resulting in a relatively high basal level of *acuI* expression. Upon effector binding, the affinity of AcuR for the promoter region is increased, thereby markedly enhancing *acuI* transcription. In contrast, the presence of these effectors significantly increases AcuR's affinity for the promoter region, thereby enhancing the transcription levels of *acuI*.

### 2.7 The Multi‐dimensional Regulation of DMSP Metabolism in Strain D47

In bacterial systems, DMSP functions as a carbon source, osmoprotectant, and anti‐stress agent. In strain D47, the toxic metabolic intermediate acryloyl‐CoA can be generated either through the action of AcuN or PrpE (a propionyl‐CoA ligase with catalytic activity for acrylate) on exogenously supplied acrylate, or via the activity of AcuK(H) homologs (which are constitutively expressed) on 3‐HP‐CoA, a byproduct of the DddD enzyme [28, 33, 35]. This intermediate is detrimental to the bacteria's survival [36, 37]. The detoxification of acryloyl‐CoA is essential due to its toxicity. Ensuring cellular physiological safety is fundamental for effective DMSP metabolism, DMS production, and nutrient acquisition.

**FIGURE 5:**
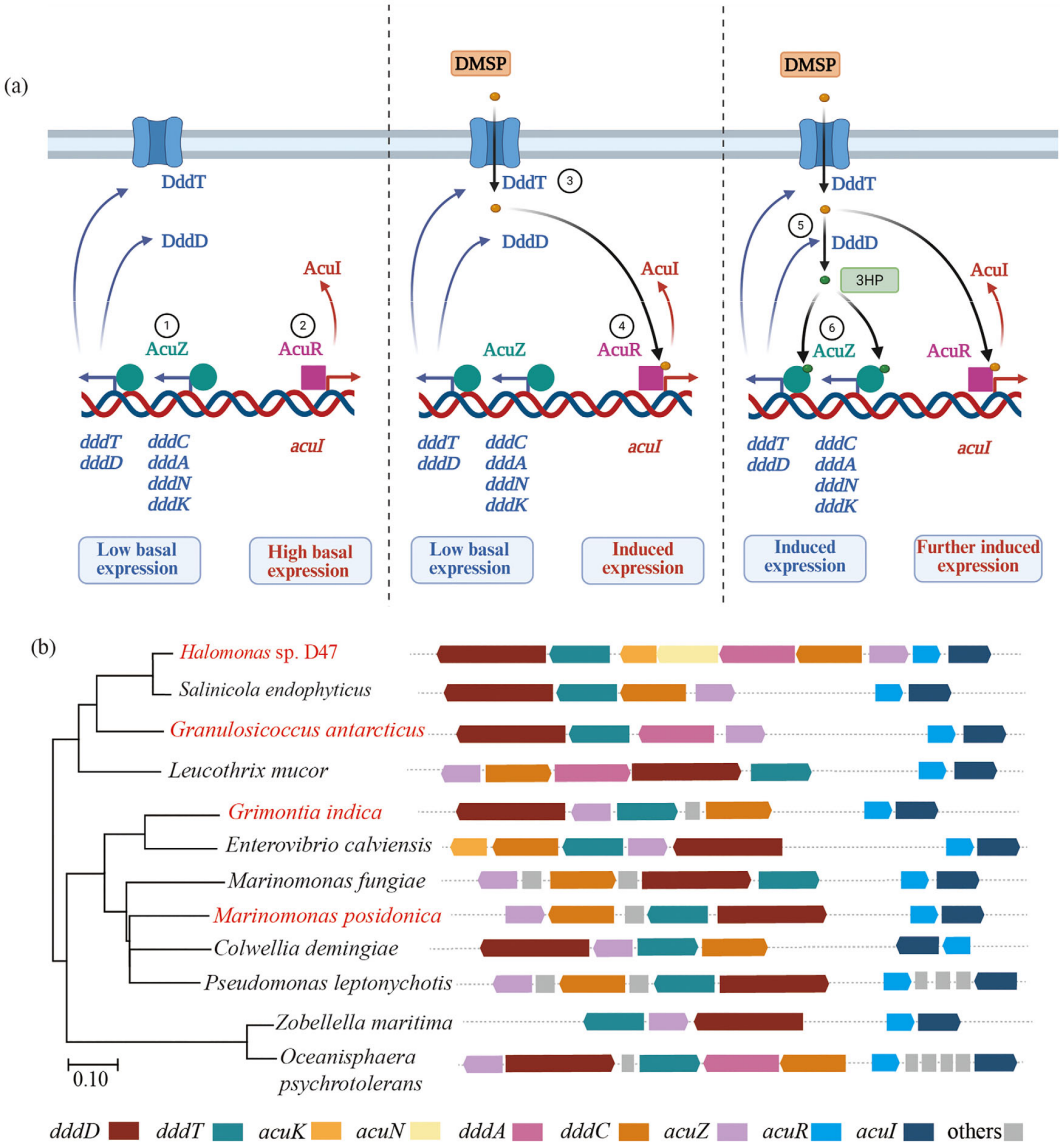
Multifaceted regulation of DMSP metabolism and its universal significance. (a) Proposed model for DMSP metabolism regulation in strain D47. ①‐② Under limited DMSP conditions, AcuZ binds to the promoters of *dddC* and *dddT*, repressing the expression of the genes involved in DMSP transport and catabolism. Meanwhile, the transcription of *acuI*, associated with detoxification, is kept at a low level through activation by AcuR. Both *acuI* and the DMSP transport and catabolism genes exhibit basal expression; however, the basal expression of *acuI* is markedly higher than that of the DMSP transport and catabolism genes. ③‐④ In the presence of environmental DMSP, DMSP can enter the cell via basally expressed DMSP transporters and act as the effector for AcuR, enhancing the affinity between AcuR and its target promoters, thereby increasing *acuI* expression. ⑤‐⑥ The active DddD enzyme cleaves intracellular DMSP into DMS and 3‐HP. The presence of 3‐HP activates the expression of genes for DMSP transport and degradation via AcuZ. At this stage, as more DMSP is imported and degraded, the entire metabolic pathway is further activated. Throughout the process, the upregulation of *acuI* expression consistently precedes the activation of DMSP degradation. (b) DMSP metabolic gene clusters with homologs for core genes across various bacterial species. These clusters are found in different groups within *γ*‐Proteobacteria and some *α*, *β*‐Proteobacteria.

At the transcriptional level, strain D47 employs sophisticated mechanisms to coordinate DMSP metabolism with the detoxification process. The primary enzymes, AcuIs, manage the toxic acryloyl‐CoA in DMSP‐catabolizing bacteria. In strain D47, AcuZ mainly regulates genes associated with DMS production and DMSP metabolism, while AcuR controls the transcription of detoxification‐related *acuI* (Figure 5a). AcuZ acts as a repressor in the absence of effectors, while AcuR functions as an activator, capable of binding to the promoter to activate *acuI* transcription regardless of effector presence. With effectors, AcuR exhibits stronger binding and more pronounced activation, maintaining a high basal expression level of *acuI* to preempt cellular toxicity.

AcuZ recognizes either 3‐HP or acrylate as its effector, while AcuR recognizes DMSP or acrylate. However, both AcuR and AcuZ have a considerably lower binding affinity for acrylate compared to 3‐HP and DMSP, respectively. These findings suggest that DMSP is the primary effector for AcuR, while 3‐HP is for AcuZ, with acrylate acting as a secondary choice for both. Acrylate's role as an alternative effector for both AcuR and AcuZ is also meaningful. Acrylate is a carbon source that occurs at nanomolar concentrations in the ocean [55]. Acting as an alternative effector, acrylate enables strain D47 to utilize environmental acrylate for growth. As shown above, strain D47 can grow with acrylate as the sole carbon source. Since DMSP is a precursor for 3‐HP, this indicates that AcuR can detect substrate signals earlier than AcuZ, enabling cells to upregulate *acuI* expression levels preemptively before toxic metabolites accumulate.

Based on these analyses, the proposed regulatory process for strain D47 is depicted in Figure 5a. In the absence of DMSP, AcuZ binds to the promoters upstream of the *dddC* and *dddT* genes, inhibiting the transcription of genes involved in DMSP transport and degradation. Meanwhile, a limited number of AcuR molecules bind weakly to the *acuI* promoter, ensuring low‐level expression of this detoxifying gene. Due to the distinct roles of AcuZ and AcuR, the transcription of DMSP transport and degradation genes remains significantly lower than the basal expression of *acuI*. When DMSP is present, it first enters the cell via the basally expressed DMSP transporters. The presence of DMSP activates AcuR, leading to stronger interactions between AcuR and its target promoters, thereby increasing the expression of *acuI*. Concurrently, the basally expressed DMSP lyases degrade DMSP into 3‐HP, which then activates AcuZ, promoting the expression of proteins involved in DMSP transport and degradation. As cells import and metabolize more DMSP into DMS and 3‐HP, there is a progressive upregulation of the related genes under AcuR and AcuZ control. Due to the mechanistic action of AcuR and its recognition of DMSP at a more upstream position than AcuZ, this sequential regulatory system ensures that *acuI* is expressed before the DMSP transport and degradation genes, thereby enabling efficient DMSP utilization while maintaining physiological integrity throughout the process.

For *R. sphaeroides* 2.4.1 and *A. faecalis* M3A, we mentioned above, the AcuRs regulate both the DMSP lyase genes and *acuI* without the presence of an AcuZ‐like repressor to modulate DMSP metabolic genes, thus lacking the fine‐tuned regulatory mechanism present in strain D47 to maintain metabolic and physiological safety balance. This may explain why the AcuRs in strain D47 and *R. sphaeroides* 2.4.1 and *A. faecalis* M3A have adopted completely opposite regulatory modes. Regulation of DMSP lyase via a repressor role contributes to the tight control of the basal expression level of the cleavage pathway, which may confer an advantage to the bacteria in terms of DMSP accumulation. Each strategy is tailored to its own unique physiological requirements. However, from the observed outcomes, the detoxification appears to be more effective in strain D47, as supported by their better growth when DMSP or acrylate is the sole carbon source.

### 2.8 Applicability of the Proposed Regulatory Mechanism

Using representative bacterial genomes from the nonredundant protein database (nr), we identified 55 putative DddD‐mediated DMSP‐catabolizing strains, mainly affiliated with the Oceanospirillaceae, Pseudomonadaceae, Halomonadaceae, and Roseobacteraceae families (Table S5 and Figure S10a). These taxa represent major contributors to marine DMSP metabolism, particularly in polar regions and marine sediments [27, 56]. Among them, the strains *Marinomonas posidonica*, *Leucothrix mucor*, and *Salinicola endophyticus* harbor DMSP catabolic gene clusters highly similar to those of strain D47 and encode AcuR and AcuZ homologs with strictly conserved residues implicated in effector binding (Figure 5b; Figure S11). Although the *acuNK* genes involved in acrylate metabolism are absent from these clusters, all these strains contain homologs of AcuH(K), which catalyzes the formation of acryloyl‐CoA during DMSP metabolism (Table S6) [35, 57].

Moreover, as observed in strain D47, genes involved in DMSP transport and degradation are frequently clustered with putative *acuZ*s, whereas putative *acuR*s are typically located in proximity to putative *acuI*s. Promoter analysis in Figure S10b further revealed that promoters of genes involved in DMSP transport and degradation contain sequences reminiscent of LysR‐family binding motifs (e.g., T‐N11‐A, ATAC‐N7‐GTAT, CTATA‐N9‐TATAG), whereas promoters of *acuI* harbor AT‐rich palindromic motifs potentially recognized by TetR‐family regulators [37, 43, 44]. Together, these genomic and regulatory features suggest that these strains employ a regulatory architecture analogous to that of strain D47, in which a LysR‐family regulator senses 3‐HP to control DMSP transport and degradation genes, while a TetR‐family regulator responds to DMSP to regulate *acuI* expression.

Some bacteria, such as *Granulosicoccus antarcticus*, also possess DddD‐mediated DMSP‐catabolizing gene clusters; however, key amino acid residues implicated in effector recognition are not fully conserved in their AcuR and AcuZ homologs (in *G. antarcticus*, AcuZ is conserved, whereas AcuR shows divergence) (Figure S11). To evaluate whether a regulatory mode related to that of strain D47 might nevertheless operate in such more divergent strains, *acuR* and *acuZ* from *G. antarcticus* were expressed and purified. Binding assays showed that the AcuR homolog binds DMSP and acrylate, whereas the AcuZ homolog binds 3‐HP and acrylate (Figure S12). These results imply that a regulatory mode resembling that of strain D47 may also operate in more divergent strains represented by *G. antarcticus*. However, given the divergence in sequence conservation, it cannot be excluded that these strains employ modified or partially distinct regulatory mechanisms. Resolving this requires further strain‐specific functional validation.

Consequently, based on the current evidence, at least the common DMSP‐degrading strains *Marinomonas posidonica*, *Leucothrix mucor*, and *Salinicola endophyticus* are highly likely to employ a regulatory mechanism for effector recognition and DMSP degradation that is closely analogous to that of strain D47.

## 3 Conclusion

In summary, our study reveals a complex regulatory mechanism in *Halomonas* sp. D47, in which the TetR‐family regulator AcuR and the LysR‐family regulator AcuZ coordinate DMSP catabolism and DMS production. These regulators integrate signals from DMSP and its metabolites, 3‐HP and acrylate, to fine‐tune gene expression, balancing metabolic activity and detoxification. Bioinformatics analyses indicate that this regulatory strategy is likely conserved across certain γ‐ Proteobacteria, suggesting a potentially bacterial mechanism for managing DMSP catabolism and maintaining cellular homeostasis.

## 4 Materials and Methods

### 4.1 Strains and Growth Conditions

All bacterial strains used in this study are listed in Table S7. Strain D47 was isolated with DMSP as the sole carbon source at 20°C from a sediment sample collected at Taipingjiao, Qingdao, China, in April 2020. The wild‐type strain D47 and its mutant derivatives were cultured at 20°C with shaking at 180 rpm in either marine Luria‐Bertani (MLB) medium, containing 10 g L^−1^ tryptone, 5 g L^−1^ yeast extract, and 30 g L^−1^ sea salt (Sigma, USA), or in a minimal medium composed of 30 g L^−1^ sea salt, 10 mM NH_4_Cl, 148 mM MgCl_2_, 13.6 mM K_2_SO_4_, 1 mM K_2_HPO_4_, 0.3 mM CaCl_2_, 22 µM FeCl_3_, 16 µM NaMoO_4_, 23 µM CuCl_2_, and 0.2 M Tris‐HCl buffer (pH 8.0). Each medium contained either DMSP, acrylate, 3‐HP, or glucose (Glc) as the sole carbon source at a concentration of 5 mM (Tokyo Chemical Industry, Japan).

### 4.2 Genome Sequencing and Analysis

Strain D47 was cultured in MLB medium at 20°C with shaking at 180 rpm until reaching an OD_600_ of 0.8. Cells were harvested by centrifugation at 8,000 × g for 5 min at 4°C, and genomic DNA was extracted from the cell pellets using the Bacteria DNA Kit (OMEGA, USA) according to the manufacturer's instructions. A DNA sample of 2 µg (OD_260/280_ = 1.8–2.0, > 6 µg) was then used to construct a fragment library for bacterial genome sequencing and assembly on the PacBio third‐generation sequencing platform at Tianjin Biochip Corporation [58]. De novo genome assembly was conducted using the HGAP 4 program of Pacbio SMRT Link (V6.0.0.47841) [59, 60]. Genome annotation was performed using RAST (Rapid Annotation using Subsystem Technology), version 59 [61]. Gene clusters involved in DMSP catabolism were identified based on genome annotation and functional annotation. Promoter regions were predicted using the PROM program (http://linux1.softberry.com).

### 4.3 Co‐transcription Analysis

Total RNA was extracted using the RNeasy Mini Kit (QIAGEN, USA). PCR was carried out on adjacent genes using cDNA template and primer pairs that spanned various intergenic regions. DNA products were only obtained when the intervening DNA between the two primers spanned the gap regions.

### 4.4 Transcriptomic Analysis

Strain D47 was cultured in MLB medium to an OD_600_ of 0.8. Cells were harvested by centrifugation at 6,500 × g for 5 min at 4°C, washed three times with 1.5 mL of 3% sea salt solution, and held at 4°C for 2 h. The cells were then inoculated (1.5% inoculum size) into media containing either DMSP or Glc, each provided at 5 mM as the sole carbon source. Samples were collected at 0 h and after approximately 15 h of incubation, by which time the cells had reached mid‐logarithmic growth phase. Cells from each sample were collected by centrifugation at 6,500 × g for 2 min at 4°C, frozen in liquid nitrogen, and sent to BIOZERON (China) for transcriptomic sequencing and analysis. Total RNA from *Halomonas* sp. D47 was extracted using TRIzol Reagent (Invitrogen) and treated with DNase I (TaKaRa) to remove genomic DNA. RNA quality was assessed (OD_260/280_ = 1.8–2.2, OD_260/230_ ≥ 2.0, RIN ≥ 6.5, 28S:18S ≥ 1.0), and >10 µg of RNA was used for library construction. cDNA libraries were size‐selected for 200–300 bp fragments using 2% Low Range Ultra Agarose, PCR‐amplified with Phusion DNA polymerase (NEB) for 15 cycles, and sequenced on the MGI‐T7 platform (PE150) by Shanghai BIOZERON Biotech Co., Ltd. Raw reads were trimmed and quality‐controlled using Trimmomatic (version 0.36). Transcript abundance was calculated as FPKM, and differential expression analysis was performed using Bioconductor edgeR.

### 4.5 Real‐time qPCR Analysis

Bacterial strains were cultured as described in the “Transcriptomic Analysis” section. Total RNA was extracted using the RNeasy Mini Kit (QIAGEN, USA). RT‐qPCR was performed using the same methods as previously described [62]. The *recA* gene was used as the reference gene. Fold change = 2‐(Sample (CTtarget‐CT*recA*)‐0 h (CTtarget‐CT*recA*)). A fold change>2 indicates significant up‐regulation; fold change<0.5 indicates significant down‐regulation. All the primers employed are listed in Table S8.

### 4.6 Construction of Recombinant Strains

The procedure for chromosomal gene knockouts in strain D47 was based on a marker‐less gene replacement method in Qin et al. [63]. To mutate acuZ, the donor homologous arms were amplified from the genome of strain D47 using primers Zup‐F/Zup‐R, Zdn‐F/Zdn‐R, and pQ1‐F/SG1‐R (Table S8). These DNA fragments were fused by overlap PCR and introduced into plasmid pSEV241 (digested with *Sac* Ι and *Not* Ι) to make plasmid pSE‐donorZ. Plasmid pQ08‐Cas9 was transformed into strain D47 first, and then pSE‐donorZ (used for specific gene editing) was introduced into the pQ08‐Cas9‐containing cells by conjugal transfer using *Escherichia coli* WM3064. Cells were grown on plates containing 70 µg mL^−1^ chloramphenicol or 50 µg mL^−1^ kanamycin. Putative deletions (termed Δ*acuZ*) were confirmed by colony PCR and sequencing. The mutant strain was sub‐cultured several times on media that lacked antibiotics and examined for any that had lost the plasmid pQ08‐Cas9.

To complement the ΔZ mutant, fragments were amplified from the genome of strain D47 using primers comZ‐F/comZ‐R (Table S8) and inserted into the plasmid pSEV241, which had been digested with *Sac* Ι and *Not* Ι, to construct the complementing plasmid pSE‐Z. The plasmid pSE‐Z was then introduced into the ΔZ mutant by conjugative transfer from *Escherichia coli* WM3064, and the phenotype of the transconjugant ΔZ‐pSEZ was examined. All mutations were confirmed by DNA sequencing. The plasmids and primers used in this experiment are listed in Tables S7 and S8.

To construct the recombinant strains BL4701, BL4702, and BL4703, DNA fragments *dddC‐dddD*, *dddZ‐dddD*, *acuI*, and *acuR‐acuI* were amplified from the strain D47 genome using primers cF/dR, ZF/dR, iF/iR, and rF/iR, respectively. DNA fragments *dddC‐dddD* and *dddZ‐dddD* were then introduced into plasmid pEV to construct plasmids pEd47‐C and pEd47‐ZC, respectively. Fragments *acuI* and *acuR‐acuI* were introduced into plasmid pBBRMCS‐1 to construct plasmids pBd47‐I and pBd47‐RI, respectively. The plasmids were transferred into *Escherichia coli* BL21 (DE3). The recombinant strains were selected by Ampicillin and Chloramphenicol, and were verified by PCR amplification. The plasmids and primers used in this experiment are listed in Tables S6 and S7.

### 4.7 Gene Cloning, Expression and Purification of the Regulatory Proteins

The nucleotide sequences of wild‐type *acuZ* and *acuR* were each PCR‐amplified from strain D47 genomic DNA and the mutant versions from pET‐acuZ and pET‐acuR, respectively, with the primers shown in Table S8 and subcloned into a pET‐22b expression plasmid (Table S7). The recombinant plasmids were transformed into *Escherichia coli* BL21 (DE3). Recombinant proteins were purified with Ni^2+^‐NTA resin (Qiagen, Germany), followed by gel filtration on a Superdex 200 10/300 GL column (GE Health‐care, USA).

### 4.8 Microscale Thermophoresis (MST) Binding Assays

The binding affinity of AcuZ and AcuR polypeptides to different effectors—DMSP, 3‐HP, and acrylate—was analyzed by microscale thermophoresis (MST). Each polypeptide was used as a target at a concentration of 10 µM, with effectors titrated in a dilution series ranging from 105 to 1.53 nM. Samples were loaded into Monolith NT.115 Pico MST standard‐treated capillaries (NanoTemper Technologies, USA) and measurements were conducted at 20°C with an excitation power setting of 50%. Fraction bound values, plotted against ligand concentration, were analyzed using the *K*_d_ model in MO Affinity Analysis software (version 2.3, NanoTemper Technologies) based on three independent experiments. The fraction bound value for each data point (ranging from 0 to 1) was calculated by dividing the ΔFnorm value of that point by the amplitude of the corresponding curve. The same method was applied to assess the binding affinity of regulator proteins to promoters.

### 4.9 Electrophoretic Mobility Shift Assays

EMSAs were conducted with purified AcuZ and AcuR proteins following the protocol described in Lu et al., 2020 [64]. Complementary biotin‐labeled oligonucleotides were used as probes in reactions containing purified protein in binding buffer (20 mM Tris‐HCl, pH 8.0; 2 mM EDTA; 20 mM KCl; 0.5 mM DTT; 4% Ficoll‐400; 50 ng µL^−^^1^ POLY(DI‐DC) (Sigma, USA)). The sequences of promoter probes used are listed in Table S3. DNA‐protein mixtures were incubated at 4°C for 30 min, then separated on 8%–15% nondenaturing polyacrylamide gels. DNA was transferred onto Immobilon‐Ny^+^ membranes (Millipore, USA) and processed according to the manufacturer's instructions. Signal detection was performed using the ECL Western Blotting Analysis System kit (GE Healthcare, USA), and bands were visualized by autoradiography (Thermo, USA).

### 4.10 Structure Prediction

The AlphaFold structures in this study were mainly generated from the AlphaFold2 implementation in the ColabFold notebooks running on Google Colaboratory [65, 66], using the default settings. PAE and pLDDT scores were computed to indicate the accuracy of a prediction.

### 4.11 Bioinformatics

To examine the distribution of the regulatory system identified in this study, the sequences of DddD and DddT from strain D47 were first used to search bacterial genomes in the representative bacterial genomes available on GenBank. The search parameters were set with an E‐value of <1E^−50^ and a similarity threshold of >35% using BLASTp. The DddD homologues were further screened based on protein sequence length, retaining only those with sequences exceeding 600 amino acids. The genomes that contain both DddT and DddD homologues were selected. Subsequently, the genomic positions of the genes corresponding to the selected protein homologues were analyzed. Only the genomes that contain *dddT* genes that are less than five genes away from *dddD* genes were retained.

### 4.12 Statistical Analysis

Data were analyzed without transformation or normalization unless otherwise stated. All quantitative data are presented as mean ± standard deviation (SD). Unless otherwise indicated, all experiments were performed using three independent biological replicates (*n* = 3). Because statistical analyses for both transcriptomic and RT–qPCR data primarily involved comparisons between two groups, statistical significance was assessed using two‐tailed statistical tests. Statistical significance is indicated by * for *p* < 0.05, ** for *p* < 0.01, and *** for *p* < 0.001. RT–qPCR data were analyzed using Origin (version 8.0), while differential gene expression from transcriptomic data was assessed using edgeR (version 4.6.3).

## Author Contributions

P.W. conceived the study. P.W., Y.‐Z.Z., and L.‐Y.Z. designed the experiments. L.‐Y.Z., X.‐M.S., W.‐X.J., P.W., L.L., Z.‐J.T., H.‐Q.W., W.‐J.Z., C.G., Q.‐L.Q., N.‐H.L., H.‐Y.C., H.‐H.F., and C.‐Y.L. conducted the experiments and data analysis. L.‐Y.Z., W.‐X.J., P.W., Y.‐Z.Z., X.‐L.C., and J.D.T. wrote and revised the manuscript. All authors contributed to the discussion and provided input to improve the manuscript.

## Funding

This work was funded by the National Key R&D Program of China (2024YFC2816000), the National Natural Science Foundation of China (32330001, 32170127, 32370136, 42206156), Program of Shandong for Taishan Scholars (tspd20240806 and tsqn202408064), Shandong Province Science Fund for Excellent Young Scholars (ZR2025QB18), Natural Science Foundation of Shandong Province Youth Branch (ZR2024QC347), and the SKLMT Frontiers and Challenges Project (SKLMTFCP‐2023‐06).

## Conflicts of Interest

The authors declare no conflicts of interest.

## Supporting information




**Supporting File**: advs74398‐sup‐0001‐SuppMat.docx.

## Data Availability

The data that support the findings of this study are available from the corresponding author upon reasonable request.
