# Supplementary material for: Metabolic Regulation of Dimethylsulfoniopropionate Cleavage and Dimethyl Sulfide Production in Halomonas sp. D47
Source: Adv Sci (Weinh). 2026 Feb 13;13(23):e14858. doi: 10.1002/advs.202514858 (PMC13104084; doi:10.1002/advs.202514858)
Supplement: Supplementary file 1 — Supporting File: advs74398‐sup‐0001‐SuppMat.docx. [file ADVS-13-e14858-s001.docx]

Supplemental Material for

**Metabolic Regulation of Dimethylsulfoniopropionate Cleavage and Dimethyl Sulfide Production in *Halomonas* sp. D47**

Li-Yuan Zheng *et al.*

*Corresponding author. Email: wangpeng3331@ouc.edu.cn (P.W.)

**This PDF file includes:**

Figure S1 to Figure S12

Table S1 to Table S8

**
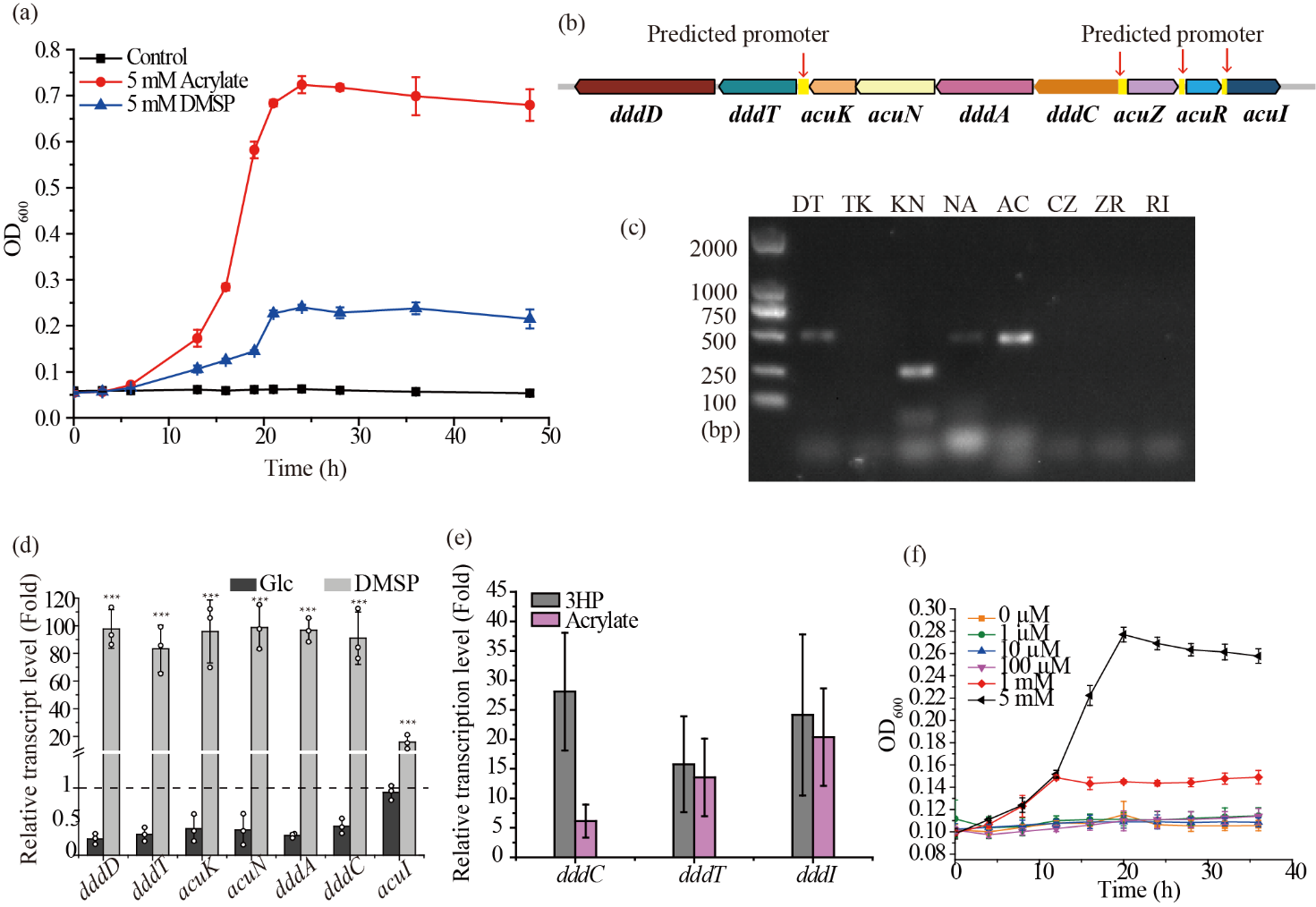
**

**Figure S1. Growth and transcriptional analysis of DMSP metabolism-related genes in strain D47.** (a) Growth curves of bacteria cultured in minimal medium supplemented with DMSP, or acrylate or with no added carbon source are shown in red, blue or black respectively. (b) Prediction of promoters within the *ddd/acu* gene cluster in strain D47 used the PROM tool from Softberry (http://linux1.softberry.com). (c) Amplification of DNA segments between genes was carried out using cDNA fragments as templates. The DNA segment between *dddD* and *dddT* is labeled as DT, with a similar naming convention applied to other segments. Note that productive PCR products were only seen in the cases of the DT, KN, NA and AC pairings. (d) RT-qPCR analysis was conducted to assess the transcription levels of genes in strain D47 when grown on 5 mM DMSP or glucose (Glc) as the sole carbon source. *p* values were compared to the corresponding glucose (Glc) group. Thus, for example, the *dddA* gene is transcribed at a level x-times higher in the DMSP-grown cells, compared to when the glucose is the sole carbon source. (e) RT-qPCR analysis was conducted to assess the transcription levels of genes in strain D47 when grown on 5 mM 3-HP or acrylate as the sole carbon source, showing that genes were also up-regulated when cultured with acrylate and 3-HP. Samples were collected at 0 hours and at the time at when the cells had reached the mid-logarithmic growth phase. (f) Growth curves of bacteria cultured with varying concentrations of DMSP. Error bars represent the standard deviation from three separate experiments. A two-tailed t-test was used for statistical comparison between different conditions. Significance levels are indicated as follows: * *p* < 0.05; ** *p* < 0.01; *** *p* < 0.001; ns denotes no significant difference (*p* ≥ 0.05).

**
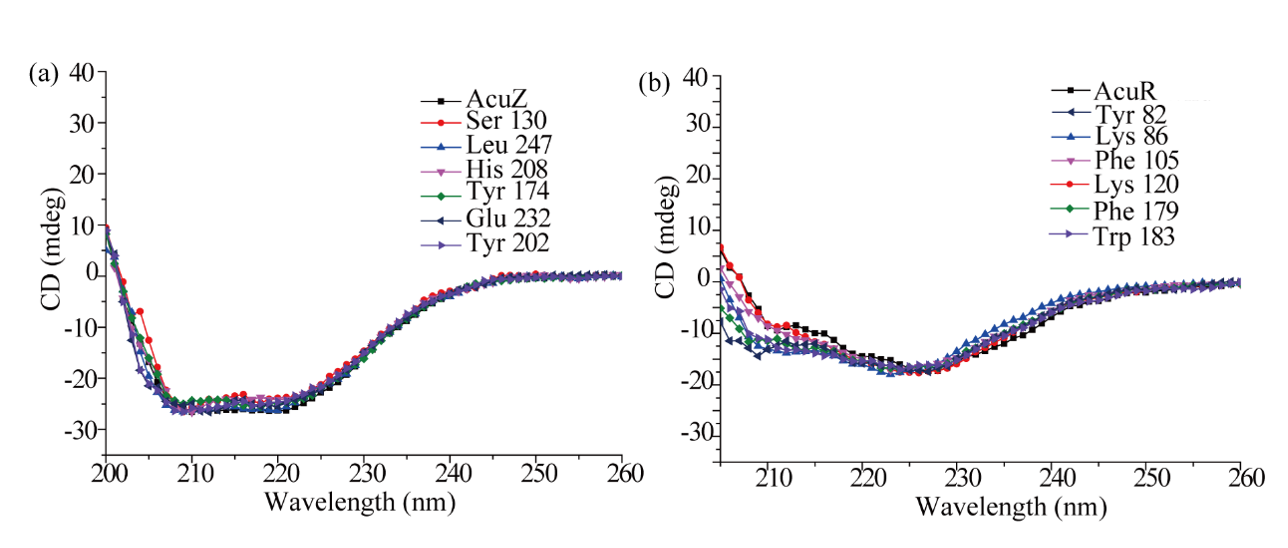
**

**Figure S2. Circular Dichroism (CD) spectra analysis of AcuZ, AcuR and their mutants.**

**
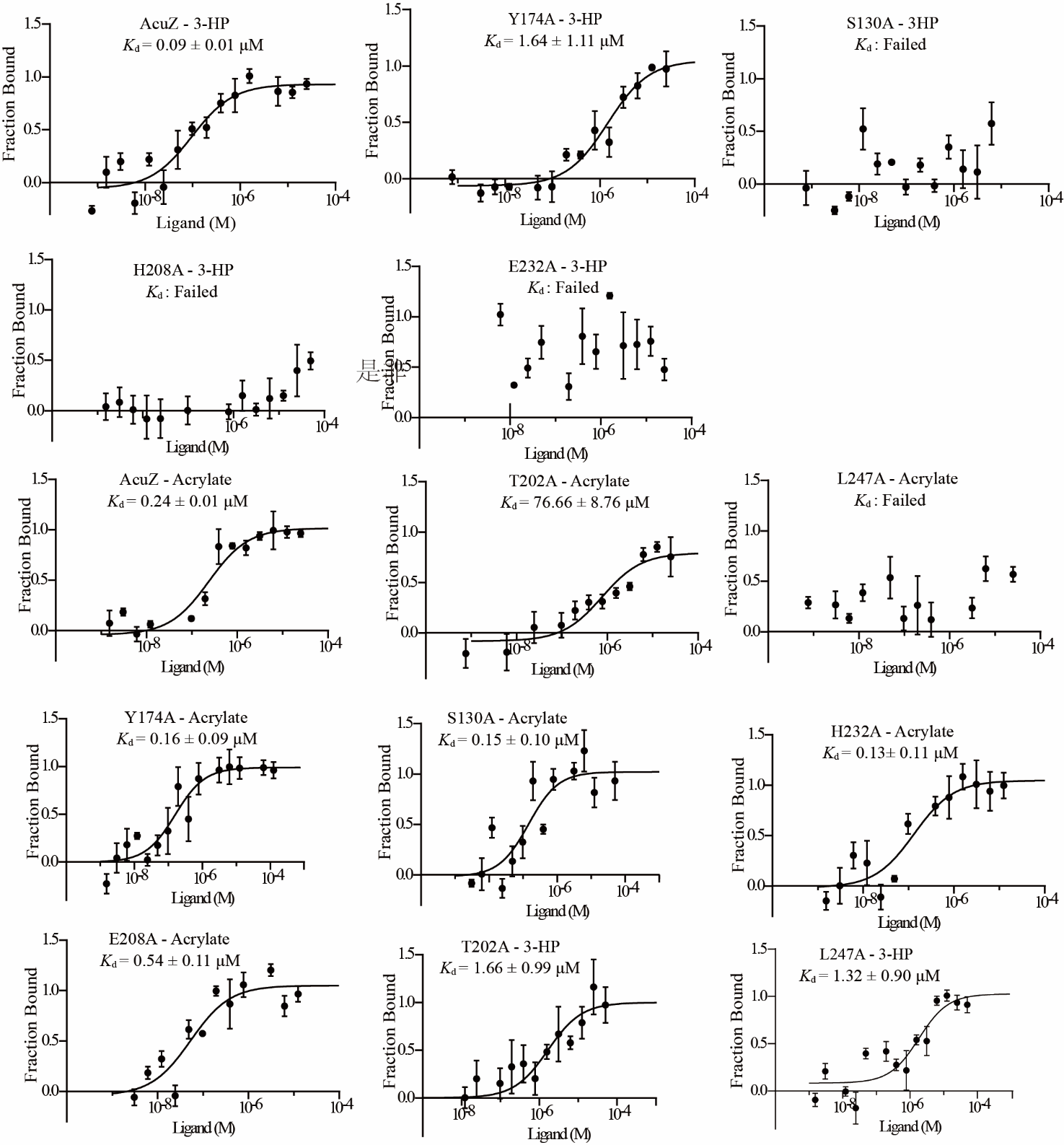
**

**Figure S3. Binding affinity analysis of AcuZ and its mutants with acrylate or 3-HP.** Data are presented as mean ± SD from three independent experiments.


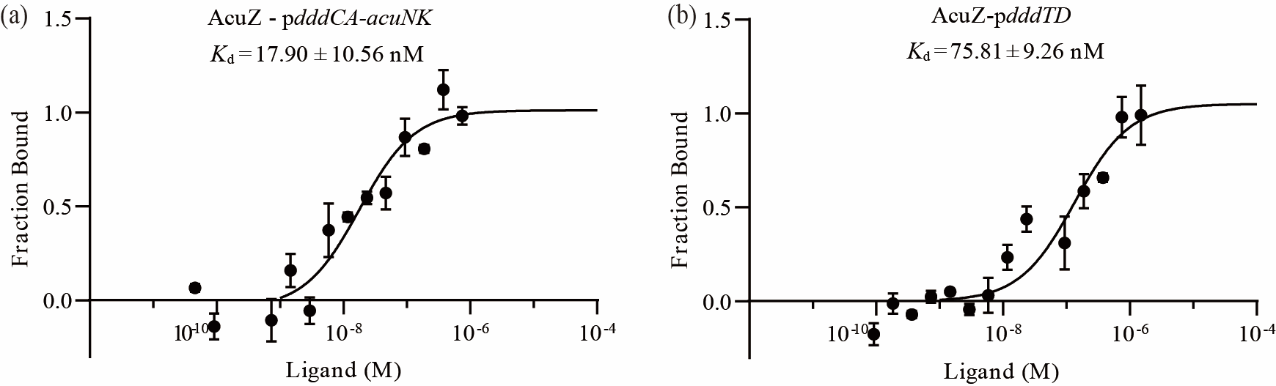


**Figure S4. AcuZ binding affinity to the promoters of *dddCA-acuNK* (a) and *dddTD* (b).** Data are presented as mean ± SD from three independent experiments.


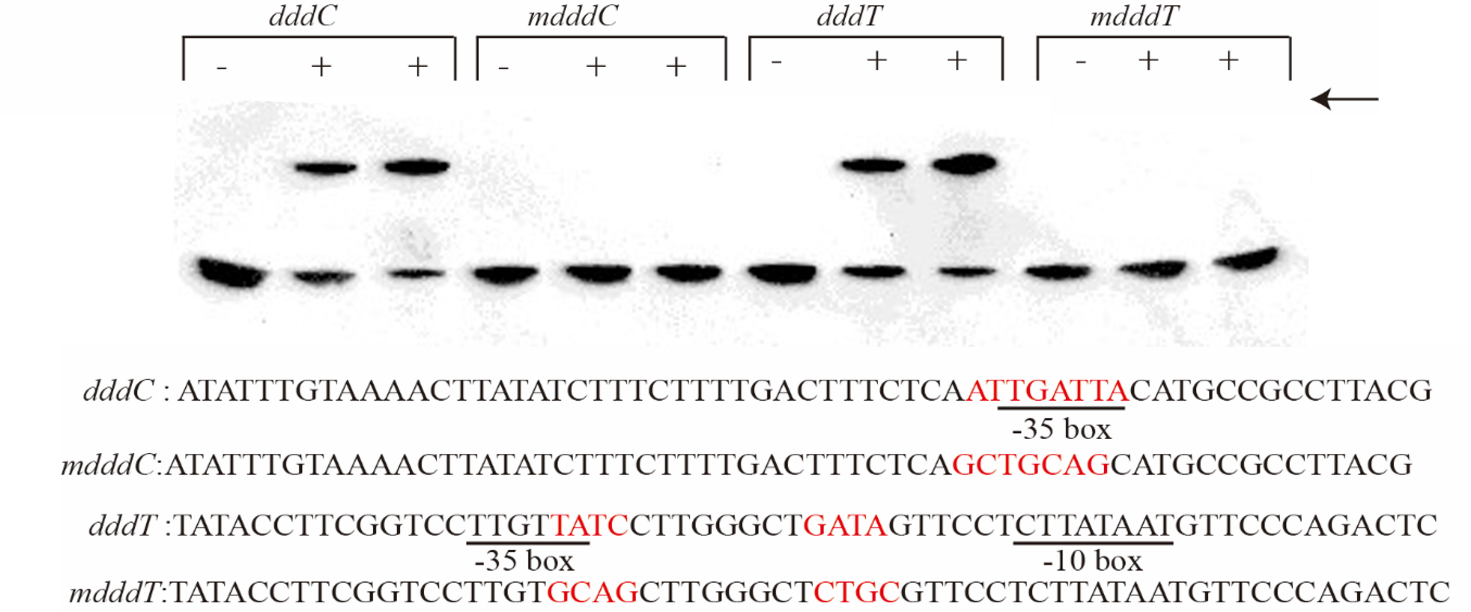


**Figure S5. EMSA analysis of AcuZ binding sites on the promoters.** Binding reactions were performed using increasing concentrations of AcuZ protein (0, 4, and 8 μM) and a fixed concentration of short oligonucleotide probes (0.2 μM). DNA probes encompassing the upstream regions of *dddC* and *dddT* were used, with the binding sites and their mutations highlighted in red.

**
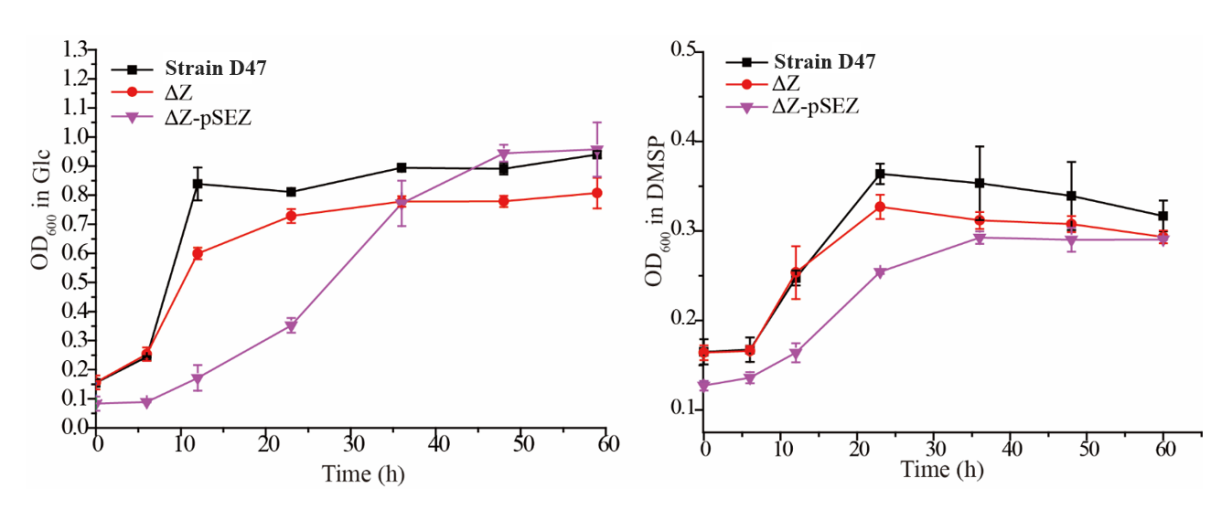
**

**Figure S6. Growth curve analysis of different mutants.** The growth curves of strain D47 and its mutants cultured using glucose (Glc) (a) or DMSP (b) as the sole carbon source. The *acuZ* deletion mutants ΔZ along with the complemented strains ΔZ-pSEZ are indicated in red and purple respectively, with wild type shown in black. Data are presented as mean ± SD from three independent experiments.

**
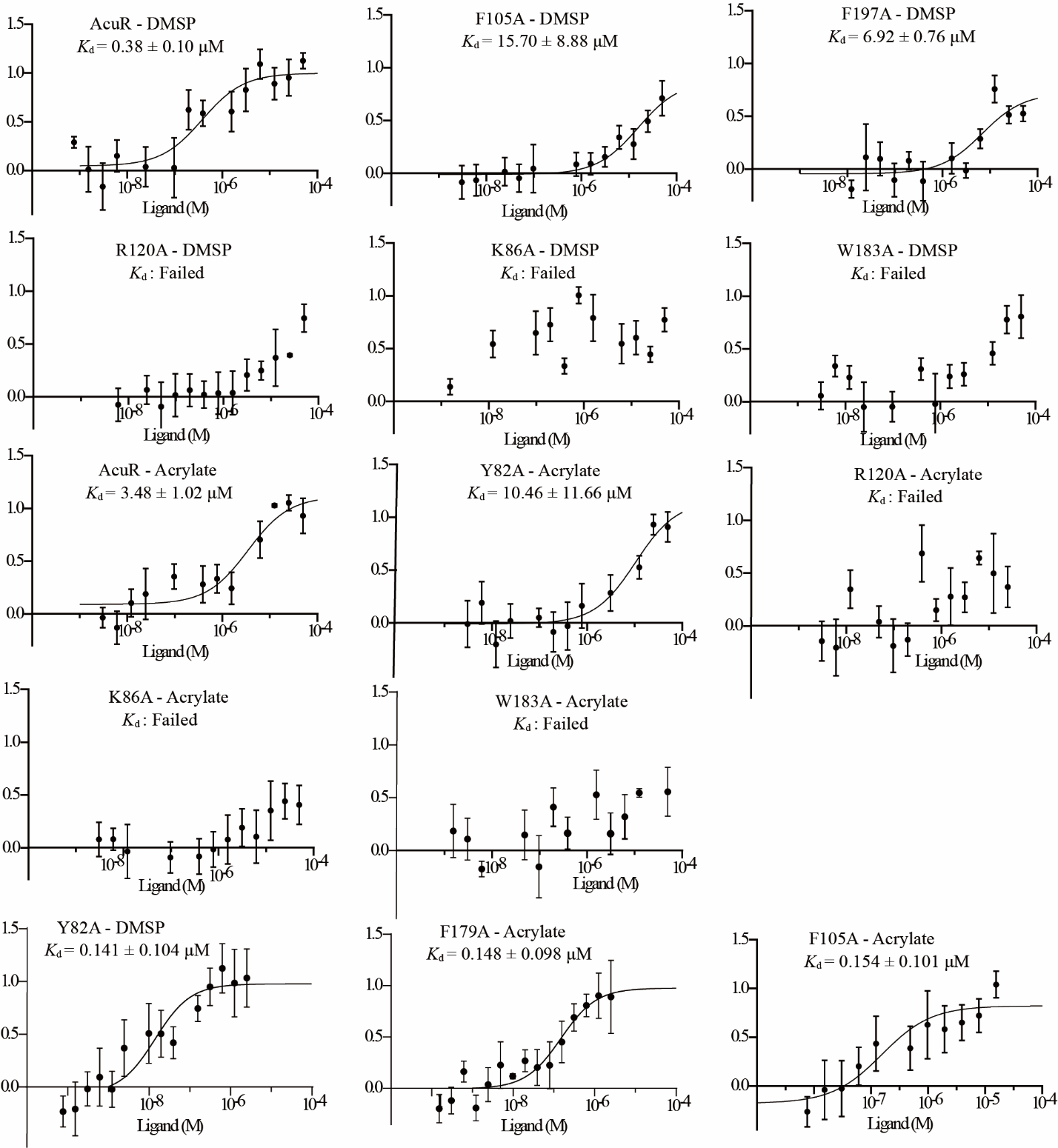
**

**Figure S7. Binding affinity analysis of AcuR and its mutants with with acrylate or DMSP.** Data are presented as mean ± SD from three independent experiments.

**
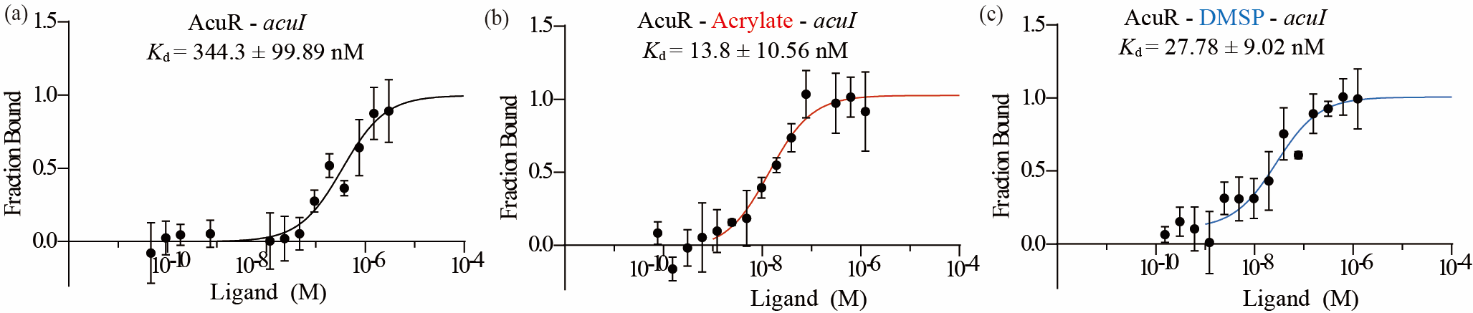
**

**Figure S8. Binding affinity analysis of AcuR with the *acuI* promoter in the presence of acrylate or DMSP.** Data are presented as mean ± SD from three independent experiments.

**
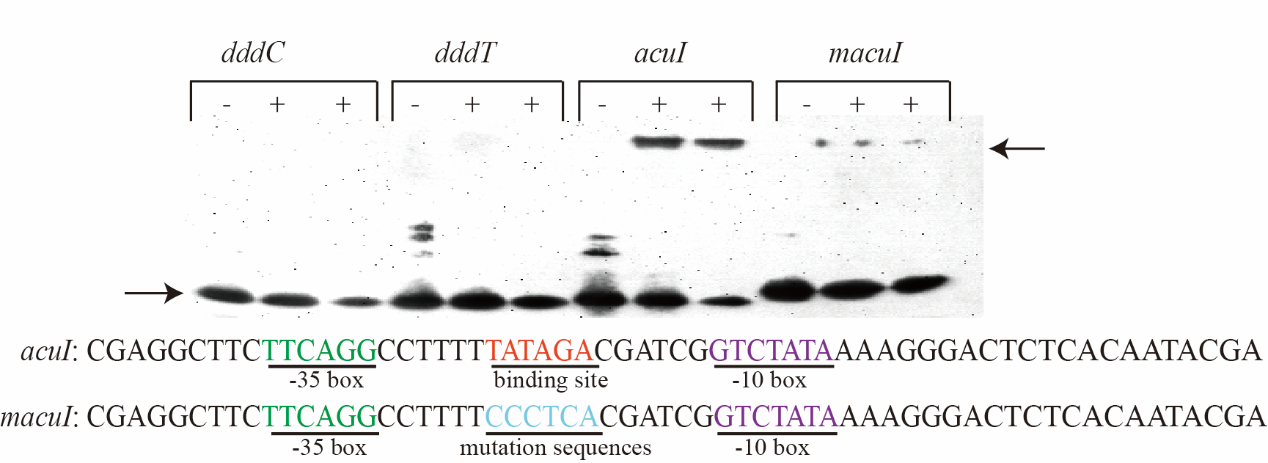
**

**Figure S9. EMSA-based analysis of AcuR binding sites on the *acuI* promoter.** Binding reactions were performed using increasing concentrations of AcuR protein (0, 10, and 20 μM) and a fixed concentration of short oligonucleotide probes (0.2 μM). The probe sequence of *acuI* promoter was present, with the proposed binding sequence and its mutations (*macuI)* was marked with red and blue color, respectively. The -35 box and -10 boxes in promoter were predicted by softberry web (http://linux1.softberry.com), and was marked with green and purple color. The predicted binding sequence is marked as a red box.

**
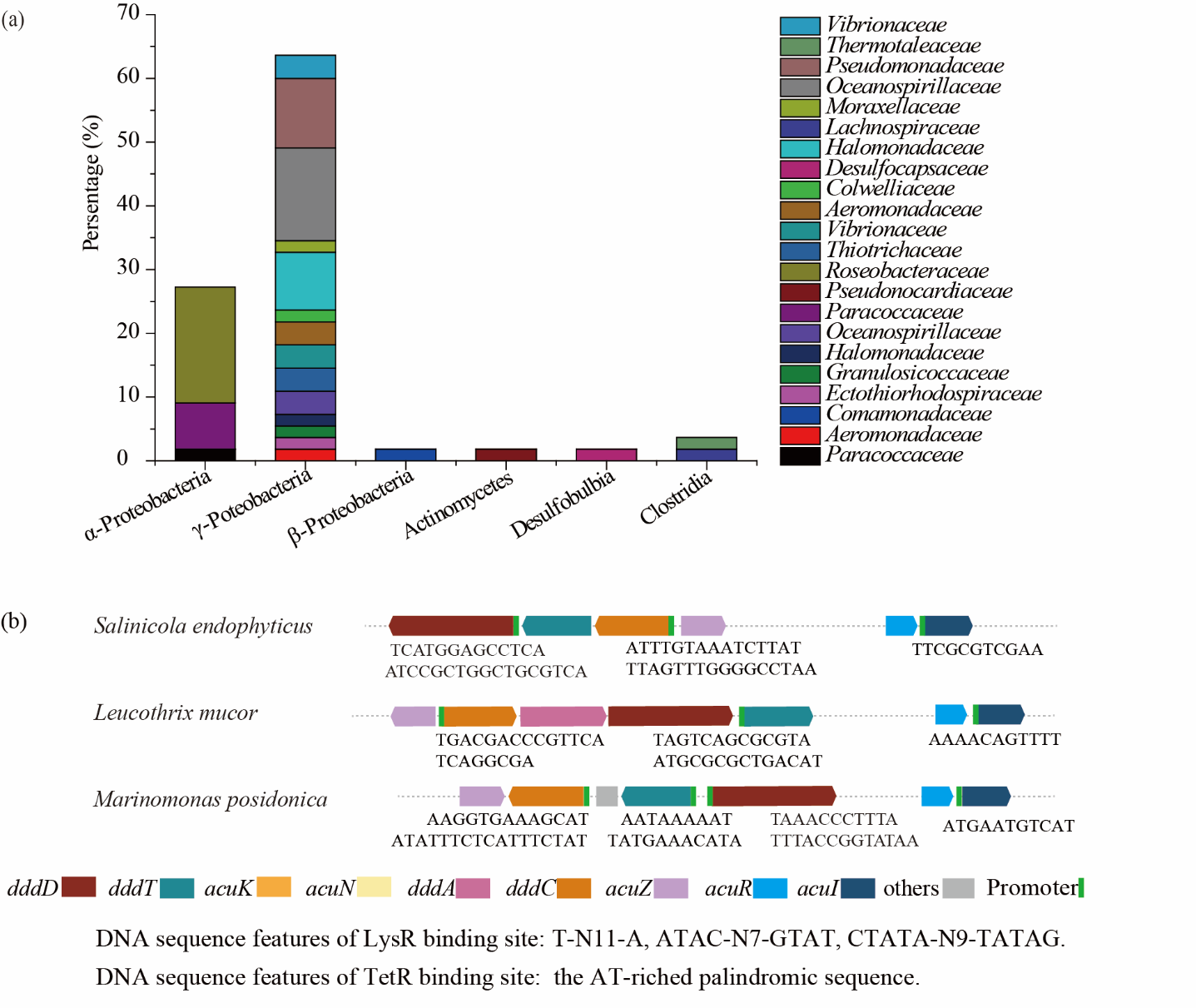
**

**Figure S10. Comparative analysis of DMSP catabolizing strains.** (a) Taxonomic distribution of 55 DMSP catabolizing strains. (b) Promoter region analysis of the three most similar strains.

**
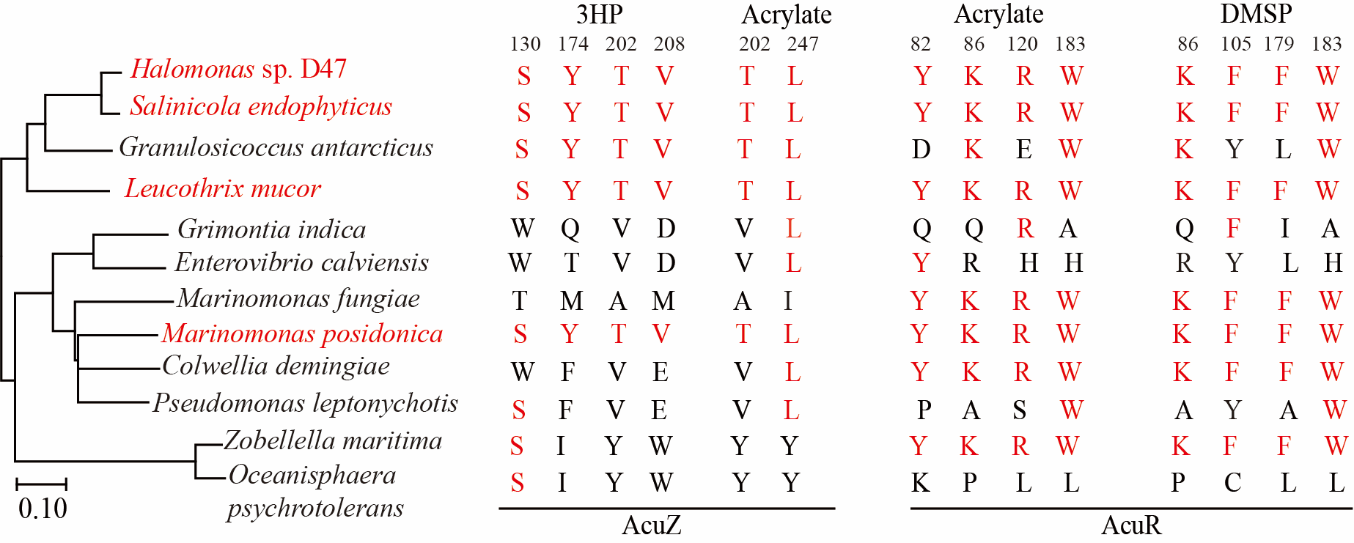
**

**Figure S11. Conservation analysis of key amino acid residues in AcuZ and AcuR homologs from different strains involved in effector recognition.** Residues identical to those in strain D47 are highlighted in red.


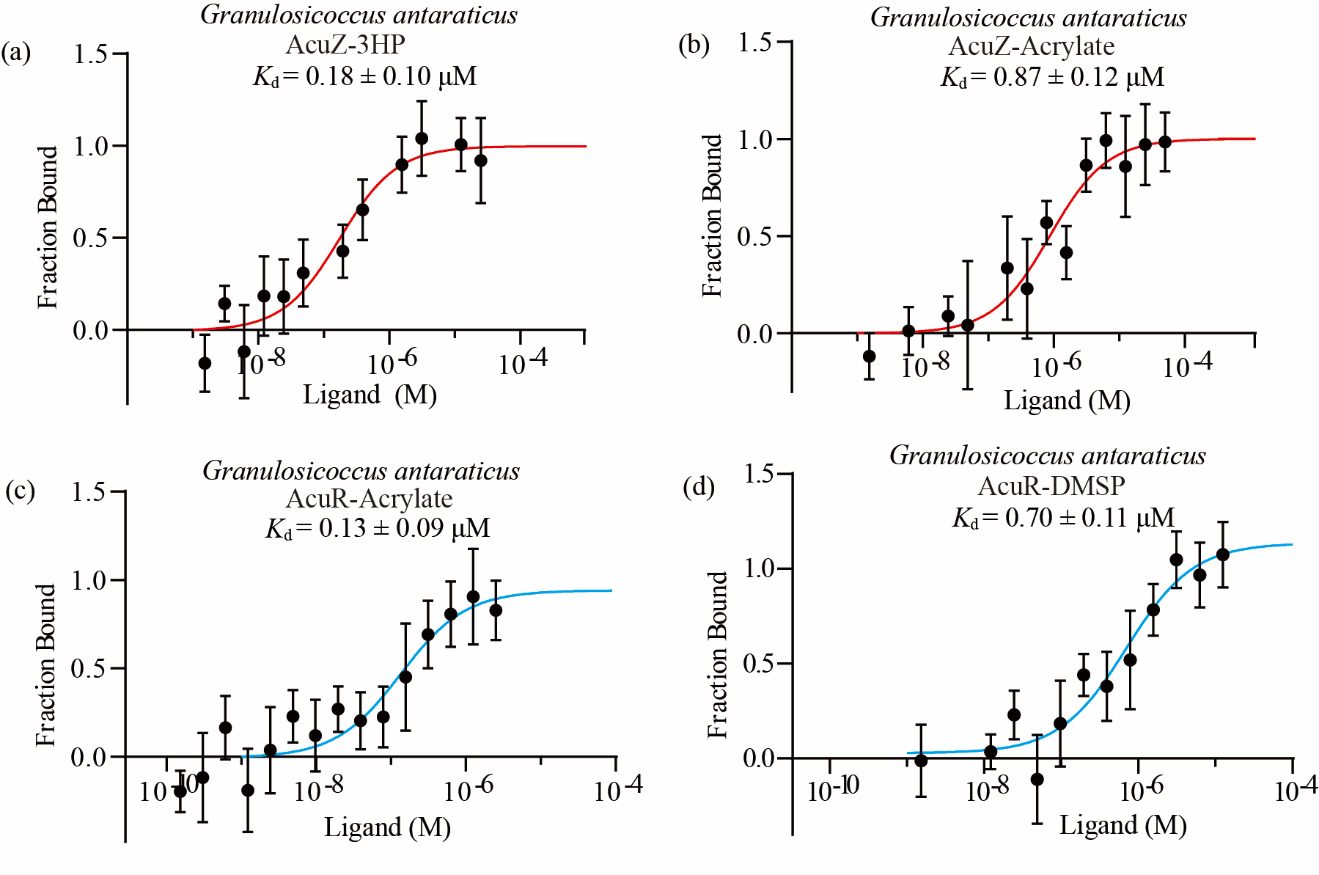


**Figure S12. Binding affinity analysis of AcuR and AcuZ homologs from *Granulosicoccus antarcticus* to DMSP, acrylate, or 3-HP.** Data are presented as mean ± SD from three independent experiments.

| \| **Table S1. Sequence identities of DMSP metabolism-related proteins between strain D47 and *Halomonas* sp. HTNK1** \| \| \| \| \| \| \| \| \| --- \| --- \| --- \| --- \| --- \| --- \| --- \| --- \| \| Protein \| Sequence ID \| % Identities \| Alignment length \| \| \| Mismatches \| E-value \| \| DddD \| ACV84065.1 \| 99% \| \| 836 \| 1 \| \| 0.0 \| \| DddT \| ACV84066.1 \| 100% \| \| 520 \| 0 \| \| 0.0 \| \| AcuK \| ACV84067.1 \| 100% \| \| 253 \| 0 \| \| 0.0 \| \| AcuN \| ACV84068.1 \| 100% \| \| 473 \| 0 \| \| 0.0 \| \| DddA \| ACV84069.1 \| 100% \| \| 579 \| 0 \| \| 0.0 \| \| DddC \| ACV84070.1 \| 99% \| \| 503 \| 1 \| \| 0.0 \| \| DddZ \| ACV84071.1 \| 100% \| \| 301 \| 0 \| \| 0.0 \| \| AcuH \| ACV84072.1 \| 100% \| \| 212 \| 0 \| \| 0.0 \| \| AcuI \| ACV84073.1 \| 99% \| \| 326 \| 1 \| \| 0.0 \|  \| Table S2. Sequence identities of AcuZ from strain D47 compared to LysR family regulators with available structures in the PDB database \| \| \| \| \| \| \| \| \| \| \| \| \| \| \| \| \| \| \| \| \| \| --- \| --- \| --- \| --- \| --- \| --- \| --- \| --- \| --- \| --- \| --- \| --- \| --- \| --- \| --- \| --- \| --- \| --- \| --- \| --- \| --- \| \| Sequence ID \| Source \| \| \| \| % Identity \| \| \| \| Alignment length in amino acid residues \| \| \| \| \| \| Mismatches \| \| \| \| E-value \| \| \| \| 3FXQ_A (TsaR) \| \| \| *Comamonas testosteroni* \| \| \| 36.559 \| \| \| \| 93 \| \| \| 55 \| \| \| \| 9.78E-08 \| \| \| \| \| \| 3M1E_A (BenM) \| \| \| *Acinetobacter baylyi ADP1* \| \| \| 35.821 \| \| \| \| 67 \| \| \| 43 \| \| \| \| 8.74E-07 \| \| \| \| \| \| 5XXP_A ( HcaR) \| \| \| *Cupriavidus necator* \| \| \| 33.803 \| \| \| \| 71 \| \| \| 47 \| \| \| \| 3.21E-04 \| \| \| \| \| \| 1IZ1_A (CbnR) \| \| \| *Cupriavidus necator* \| \| \| 29.452 \| \| \| \| 146 \| \| \| 99 \| \| \| \| 6.74E-07 \| \| \| \| \| \| 1IXC_A (catM) \| \| \| *Cupriavidus necator* \| \| \| 29.452 \| \| \| \| 146 \| \| \| 99 \| \| \| \| 1.85E-05 \| \| \| \| \| \| 2H98_A (BenM) \| \| \| *Acinetobacter baylyi* ADP1 \| \| \| 28.472 \| \| \| \| 144 \| \| \| 102 \| \| \| \| 3.70E-09 \| \| \| \| \| \| 3K1P_A (BenM) \| \| \| *Acinetobacter baylyi* ADP1 \| \| \| 27.891 \| \| \| \| 147 \| \| \| 99 \| \| \| \| 4.34E-08 \| \| \| \| \| \| 3K1N_A (BenM) \| \| \| *Acinetobacter baylyi* ADP1 \| \| \| 27.891 \| \| \| \| 147 \| \| \| 99 \| \| \| \| 4.34E-08 \| \| \| \| \| \| 2H99_A (BenM) \| \| \| *Acinetobacter baylyi* ADP1 \| \| \| 27.891 \| \| \| \| 147 \| \| \| 99 \| \| \| \| 4.62E-08 \| \| \| \| \| \| 3K1M_A (BenM) \| \| \| *Acinetobacter baylyi* ADP1 \| \| \| 27.891 \| \| \| \| 147 \| \| \| 99 \| \| \| \| 4.80E-08 \| \| \| \| \| \| **Table S3. Sequences of the promoters and the promoter probes**   \| Name \| Sequences \| \| --- \| --- \| \| *dddC* promoter \| CGCCTTAAGGTCATGCATGAATGCCTCCTTACAAATGATCGGTTCATAGAGCGTCAAGATATTTGTAAAACTTATATCTTTCTTTTGACTTTCTCAATTGATTACATGCCGCCTTACGTTAGTCTTGAGCCGACAGAGCCCTAGTGTATAGGGCTTTAAAAGCTGCAGTGCAACATAAGAAGAGAGGCAGGT \| \| *dddC* promoter probes \| ATATTTGTAAAACTTATATCTTTCTTTTGACTTTCTCAATTGATTACATGCCGCCTTACG \| \| *dddT* promoter \| ATATATTCAAAGCCCTGTCAATAAACCTAGAAAAAACAAATATCAACAACTAATAGGTAGATAACATGAACACGCCAAACAAGCCTGGCGATAGCATGGATTTAAGGGTTTTTATACCTTCGGTCCTTGTTATCCTTGGGCTGATAGTTCCTCTTATAATGTTCCCAGACTCGGGGACTCTCCTGGTTAATGCCGCTTTCGCCTTTGCAACCGGAAATTTCGGTTGGTTATAT \| \| *dddT* promoter probes \| TATACCTTCGGTCCTTGTTATCCTTGGGCTGATAGTTCCTCTTATAATGTTCCCAGACTC \| \| *acuI* promoter \| GGCTTGCCCCGCTAAACGAGGCTTCTTCAGGCCTTTTACGAGGCTTCTTCAGGCCTTTTTATAGACGATCGGTCTATAAAAGGGACTCTCACAATACGAGGTGCTAC \| \| *acuI* promoter probes \| CGAGGCTTCTTCAGGCCTTTTACGAGGCTTCTTCAGGCCTTTTTATAGACGATCGGTCTATAAAAGGGACTCTCACAATACGA \|   Table S4. Sequence identities of AcuR from strain D47 compared to TetR family regulators with available structures in the PDB database \| \| \| \| \| \| \| \| \| \| \| \| \| \| \| \| \| \| \| \| \| \| \| \| Sequence ID \| \| Source \| \| \| \| \| % Identity \| \| \| \| Alignment length \| \| \| \| \| Mis-  matches \| \| \| \| E value \| \| \| \| 3BRU_A (AcuR) \| \| *Cereibacter sphaeroides* 2.4.1 \| \| \| \| \| \| 54.75 \| \| \| \| 179 \| \| 81 \| \| \| \| 1.36E-61 \| \| \| \| \| \| 6ZUI_A (NemR) \| \| *Escherichia coli* K-12 \| \| \| \| \| 51.67 \| \| \| \| \| 60 \| \| 28 \| \| \| \| 4.86E-11 \| \| \| \| \| \| 5XAZ_A (EnvR) \| \| *Streptomyces fradiae* \| \| \| \| \| 40.39 \| \| \| \| \| 52 \| \| 31 \| \| \| \| 5.40E-05 \| \| \| \| \| \| 5XAY_A (EnvR) \| \| *Streptomyces fradiae* \| \| \| \| \| 40.39 \| \| \| \| \| 52 \| \| 31 \| \| \| \| 6.39E-05 \| \| \| \| \| \| 3RD3_A (Pa2196) \| \| *Pseudomonas aeruginosa* \| \| \| \| \| \| 33.52 \| \| \| \| 182 \| \| 120 \| \| \| \| 4.70E-23 \| \| \| \| \| \| 4L62_A (Pa2196) \| \| *Pseudomonas aeruginosa* \| \| \| \| \| \| 33.52 \| \| \| \| 182 \| \| 120 \| \| \| \| 5.21E-23 \| \| \| \| \| \| 3GEU_A (IcaR) \| \| \| \| *Staphylococcus aureus* \| \| \| 33.33 \| \| \| \| \| 45 \| \| 30 \| \| \| \| 0.001 \| \| \| \| \| \| 4YZE_A (NemR) \| \| \| \| *Escherichia coli* K-12 \| \| \| 32.77 \| \| \| \| \| 177 \| \| 117 \| \| \| \| 1.74E-19 \| \| \| \| \| \| 6AYI_A (UidR) \| \| \| \| *Escherichia coli* O157:H7 \| \| \| 32.70 \| \| \| \| \| 52 \| \| 35 \| \| \| \| 1.54E-04 \| \| \| \| \| \| 6G87_A (UidR) \| \| \| \| *Bradyrhizobium diazoefficiens* \| \| \| \| 29.79 \| \| \| \| 141 \| \| 83 \| \| \| \| 3.05E-07 \| \| \| \| \| \| 6G8H_A (UidR) \| \| \| \| *Bradyrhizobium diazoefficiens* \| \| \| \| 29.79 \| \| \| \| 141 \| \| 83 \| \| \| \| 3.37E-07 \| \| \| \| \| \| 3DPJ_A \| \| \| \| *Ruegeria pomeroyi* \| \| \| 29.71 \| \| \| \| \| 138 \| \| 78 \| \| \| \| 7.23E-08 \| \| \| \| \| \| 4UDS_A (MbdR) \| \| \| \| *Azoarcus* sp. CIB \| \| \| 28.38 \| \| \| \| \| 74 \| \| 53 \| \| \| \| 1.49E-05 \| \| \| \| \| \| 3KNW_A \| \| \| \| *Acinetobacter baylyi* ADP1 \| \| \| 27.68 \| \| \| \| \| 177 \| \| 122 \| \| \| \| 1.54E-15 \| \| \| \| \| \| 6WPA_A (AvaR1) \| \| \| \| *Streptomyces avermitilis* \| \| \| 26.90 \| \| \| \| \| 171 \| \| 112 \| \| \| \| 2.82E-09 \| \| \| \| \| \| 6WP9_A (AvaR1) \| \| \| \| *Streptomyces avermitilis* \| \| \| 26.90 \| \| \| \| \| 171 \| \| 112 \| \| \| \| 3.14E-09 \| \| \| \| \| \| 6WP7_A (AvaR1) \| \| \| \| *Streptomyces avermitilis* \| \| \| 26.90 \| \| \| \| \| 171 \| \| 112 \| \| \| \| 3.49E-09 \| \| \| \| \| \| 1SGM_A (yxaF) \| \| *Bacillus subtilis* \| \| \| \| \| 24.50 \| \| \| \| \| 151 \| \| 105 \| \| \| \| 6.32E-09 \| \| \| \| \| \| 3CCY_A \| \| *Bordetella parapertussis* \| \| \| \| \| 24.19 \| \| \| \| \| 124 \| \| 83 \| \| \| \| 0.024 \| \| \| \| \|   **Table S5.** **Strains containing DddD-mediated DMSP catabolism in the non-redundant protein database** | |
| --- | --- | --- | --- | --- | --- | --- | --- | --- | --- | --- | --- | --- | --- | --- | --- | --- | --- | --- | --- | --- | --- | --- | --- | --- | --- | --- | --- | --- | --- | --- | --- | --- | --- | --- | --- | --- | --- | --- | --- | --- | --- | --- | --- | --- | --- | --- | --- | --- | --- | --- | --- | --- | --- | --- | --- | --- | --- | --- | --- | --- | --- | --- | --- | --- | --- | --- | --- | --- | --- | --- | --- | --- | --- | --- | --- | --- | --- | --- | --- | --- | --- | --- | --- | --- | --- | --- | --- | --- | --- | --- | --- | --- | --- | --- | --- | --- | --- | --- | --- | --- | --- | --- | --- | --- | --- | --- | --- | --- | --- | --- | --- | --- | --- | --- | --- | --- | --- | --- | --- | --- | --- | --- | --- | --- | --- | --- | --- | --- | --- | --- | --- | --- | --- | --- | --- | --- | --- | --- | --- | --- | --- | --- | --- | --- | --- | --- | --- | --- | --- | --- | --- | --- | --- | --- | --- | --- | --- | --- | --- | --- | --- | --- | --- | --- | --- | --- | --- | --- | --- | --- | --- | --- | --- | --- | --- | --- | --- | --- | --- | --- | --- | --- | --- | --- | --- | --- | --- | --- | --- | --- | --- | --- | --- | --- | --- | --- | --- | --- | --- | --- | --- | --- | --- | --- | --- | --- | --- | --- | --- | --- | --- | --- | --- | --- | --- | --- | --- | --- | --- | --- | --- | --- | --- | --- | --- | --- | --- | --- | --- | --- | --- | --- | --- | --- | --- | --- | --- | --- | --- | --- | --- | --- | --- | --- | --- | --- | --- | --- | --- | --- | --- | --- | --- | --- | --- | --- | --- | --- | --- | --- | --- | --- | --- | --- | --- | --- | --- | --- | --- | --- | --- | --- | --- | --- | --- | --- | --- | --- | --- | --- | --- | --- | --- | --- | --- | --- | --- | --- | --- | --- | --- | --- | --- | --- | --- | --- | --- | --- | --- | --- | --- | --- | --- | --- | --- | --- | --- | --- | --- | --- | --- | --- | --- | --- | --- | --- | --- | --- | --- | --- | --- | --- | --- | --- | --- | --- | --- | --- | --- | --- | --- | --- | --- | --- | --- | --- | --- | --- | --- | --- | --- | --- | --- | --- | --- | --- | --- | --- | --- | --- | --- | --- | --- | --- | --- | --- | --- | --- | --- | --- | --- | --- | --- | --- | --- | --- | --- | --- | --- | --- | --- | --- | --- | --- | --- | --- | --- | --- | --- | --- | --- | --- | --- | --- | --- | --- | --- | --- | --- | --- | --- | --- | --- | --- | --- | --- | --- | --- | --- | --- | --- | --- | --- | --- | --- | --- | --- | --- | --- | --- | --- | --- | --- | --- | --- | --- | --- | --- | --- | --- | --- | --- | --- | --- | --- | --- | --- | --- | --- | --- | --- | --- | --- | --- | --- | --- | --- | --- | --- | --- | --- | --- | --- | --- | --- | --- | --- | --- | --- | --- | --- | --- | --- | --- | --- | --- | --- | --- | --- | --- | --- | --- | --- | --- | --- | --- | --- | --- | --- | --- | --- | --- | --- | --- | --- | --- | --- | --- | --- | --- | --- | --- | --- | --- | --- | --- | --- | --- | --- | --- | --- | --- | --- | --- | --- | --- | --- | --- | --- | --- | --- | --- | --- | --- | --- | --- | --- | --- | --- | --- | --- | --- | --- | --- | --- | --- | --- | --- | --- | --- | --- | --- | --- | --- | --- | --- | --- | --- | --- | --- | --- | --- | --- | --- | --- | --- | --- | --- | --- | --- | --- | --- | --- | --- | --- | --- | --- | --- | --- | --- | --- | --- | --- | --- | --- | --- | --- | --- | --- | --- | --- | --- | --- | --- | --- | --- | --- | --- | --- | --- | --- | --- | --- | --- | --- | --- | --- | --- | --- | --- | --- | --- | --- | --- | --- | --- | --- | --- | --- | --- | --- | --- | --- | --- | --- | --- | --- | --- | --- | --- | --- | --- | --- | --- | --- | --- | --- | --- | --- | --- | --- | --- | --- | --- | --- | --- | --- | --- | --- | --- | --- | --- | --- | --- | --- | --- | --- | --- | --- | --- | --- | --- | --- | --- | --- | --- | --- | --- | --- | --- | --- | --- | --- | --- | --- | --- | --- | --- | --- | --- | --- | --- | --- | --- | --- | --- | --- | --- | --- | --- | --- | --- | --- | --- | --- | --- | --- | --- | --- | --- | --- | --- | --- | --- | --- | --- | --- | --- | --- | --- | --- | --- | --- | --- | --- | --- | --- | --- | --- | --- | --- | --- | --- | --- | --- | --- | --- | --- | --- | --- | --- | --- | --- | --- | --- | --- | --- | --- | --- | --- | --- | --- | --- | --- | --- | --- | --- | --- | --- | --- | --- | --- | --- | --- | --- | --- | --- | --- | --- | --- | --- | --- | --- | --- | --- | --- | --- | --- | --- | --- | --- | --- | --- | --- | --- | --- | --- | --- | --- | --- | --- | --- | --- | --- | --- | --- | --- | --- | --- | --- | --- | --- | --- | --- | --- | --- | --- | --- | --- | --- | --- | --- | --- | --- | --- | --- | --- | --- | --- | --- | --- | --- | --- | --- | --- | --- | --- | --- | --- | --- | --- | --- | --- | --- | --- | --- | --- | --- | --- | --- | --- | --- | --- | --- | --- | --- | --- | --- | --- | --- | --- | --- | --- | --- | --- | --- | --- | --- | --- | --- | --- | --- | --- | --- | --- | --- | --- | --- | --- | --- | --- | --- | --- | --- | --- | --- | --- | --- | --- | --- | --- | --- | --- | --- | --- | --- | --- | --- | --- |
| Genome ID | Bacteria |
| GCF_004803475.1 | *Aliishimia ponticola* |
| GCF_014652655.1 | *Amylibacter ulvae* |
| GCF_002270055.1 | *Anaeromicrobium sediminis* |
| GCF_003254175.1 | *Celeribacter halophilus* |
| GCF_020217465.1 | *Cobetia amphilecti* |
| GCF_029846315.1 | *Cobetia litoralis* |
| GCF_009931455.1 | *Cobetia pacifica* |
| GCF_007954275.1 | *Colwellia demingiae* |
| GCF_900104445.1 | *Desulforhopalus singaporensis* |
| GCF_004120195.1 | *Enterovibrio baiacu* |
| GCF_000621165.1 | *Enterovibrio calviensis* |
| GCF_900537165.1 | *Enterovibrio norvegicus* |
| GCF_900184895.1 | *Flavimaricola marinus* |
| GCF_002215215.1 | *Granulosicoccus antarcticus* |
| GCF_000333895.2 | *Grimontia indica* |
| GCF_003045775.1 | *Halomonas* |
| GCF_000409775.1 | *Halomonas anticariensis* |
| GCF_000265245.1 | *Halomonas smyrnensis* |
| GCF_001761385.1 | *Hydrogenophaga crassostreae* |
| GCF_000473165.1 | *Leisingera aquimarina* |
| GCF_000511355.1 | *Leisingera methylohalidivorans* |
| GCF_025857195.1 | *Leisingera* sp. BMJM1 |
| GCF_003172895.1 | *Leucothrix arctica* |
| GCF_000419525.1 | *Leucothrix mucor* |
| GCF_014195545.1 | *Limimaricola variabilis* |
| GCF_001651805.1 | *Marinobacterium aestuarii* |
| GCF_019795155.1 | *Marinobacterium arenosum* |
| GCF_014805825.1 | *Marinomonas Algicola* |
| GCF_003314975.1 | *Marinomonas aquiplantarum* |
| GCF_001418205.1 | *Marinomonas fungiae* |
| GCF_900089985.1 | *Marinomonas gallaica* |
| GCF_003362755.1 | *Marinomonas piezotolerans* |
| GCF_000214215.1 | *Marinomonas posidonica* |
| GCF_003721275.1 | *Marinomonas rhizomae* |
| GCF_002242685.1 | *Oceanimonas* |
| GCF_001870485.1 | *Oceanisphaera psychrotolerans* |
| GCF_003208435.1 | *Pelagimonas varians* |
| GCF_029961155.1 | *Pontibacterium granulatum* |
| GCF_900116005.1 | *Poseidonocella sedimentorum* |
| GCF_009835145.1 | *Profundibacterium mesophilum* |
| GCF_900100795.1 | *Pseudomonas abietaniphila* |
| GCF_018704125.1 | *Pseudomonas boanensis* |
| GCF_900113745.1 | *Pseudomonas guineae* |
| GCF_018398425.1 | *Pseudomonas lalucatii* |
| GCF_004920405.1 | *Pseudomonas leptonychotis* |
| GCF_014269185.2 | *Pseudomonas oryzicola* |
| GCF_006716445.1 | *Pseudonocardia kunmingensis* |
| GCF_904846105.1 | *Psychrobacter glaciei* |
| GCF_900172355.1 | *Roseisalinus antarcticus* |
| GCF_019218285.1 | *Roseobacteraceae* |
| GCF_012932215.1 | *Roseobacterponti* |
| GCF_003206575.1 | *Salinicola* |
| GCF_009649225.1 | *Spiribacter salilacus* |
| GCF_003058085.1 | *Yoonia sediminilitoris* |
| GCF_003075035.1 | *Zobellella maritima* |

| **Table S6. Amino acid Sequence identities of AcuH homologs in different strains compared to functionally validated** **AcuH from *Ruegeria pomeroyi* DSS-3** | | | |  |
| --- | --- | --- | --- | --- |
| Strain | Gene ID* | e-value | Identities |  |
| *Halomonas* sp. D47 | S-A002974 | 1.82e-93 | 97/205(47%) |  |
| *Halomonas* sp. HTNK1 | ACV84067.1 | 1.82e-93 | 97/205(47%) |  |
| *Salinicola endophyticus* | WP_110674214.1 | 3e-22 | 79/167(47%) |  |
| *Marinomonas posidonica* | WP_013795567.1 | 2e-61 | 150/245(61%) |  |
| *Leucothrix mucor* | WP_022953371.1 | 1e-28 | 132/258(51%) |  |

| **Table S7. Plasmids and strains used in this study** | | |
| --- | --- | --- |
| Plasmid or strain | Genotype (descriptions) | Source or reference |
| pSEVA241 | oriV Pro1600/ColE1, oriT RP4, Kanaᴿ | From Qin et al., 2018^[1]^ |
| pQ08 | pSEVA321 derivative, *S. pyogenes cas9*, Cmᴿ | From Qin et al., 2018^[1]^ |
| pSE-donorZ | pSEVA241 derivative, sgRNA (*acuZ*),  △*acuZ* donor, Kanaᴿ | From this study |
| pSE-*Z* | pSEVA321 derivative, *acuZ,* Cmᴿ. | From this study |
| pET22b | pBR322 ori, T7p, C-terminal 6xHis, Ampᴿ. | From Novagen, Germany |
| pET-*acuZ* | PET22b derivative, *acuZ,* Ampᴿ. | From this study |
| pET-*acuR* | pET22b derivative, *acuR*, Ampᴿ. | From this study |
| pEV | pBR322 ori, T7p, *lac*p, Cmᴿ, Ampᴿ. | From Tang et al., 2020^[2]^ |
| pEd47-C | pEV derivative, *dddC*, *dddA,* *acuN*, acuK, *dddT,* *dddD,* Ampᴿ. | From this study |
| pEd47-CZ | pEV derivative, *acuZ,* *dddC*, *dddA,* *acuN*, acuK, *dddT,* *dddD,* Ampᴿ. | From this study |
| pBBRMCS-1 | IncA/C broad-host-range cloning vector, lacZα-MCS, mob, T7p, T3p, Cmᴿ. | From Kovach.M et al., 1994^[3]^ |
| pBd47-I | pBBRMCS-1derivative, *acuI*, Cmᴿ. | From this study |
| pBd47-RI | pBBRMCS-1 derivative, *acuR, acuI*, Cmᴿ. | From this study |
| *Halomonas*  sp. D47 | Wild type and used for studying the metabolic regulation of DMSP. | From this study |
| *Escherichia*  *coli* DH5α： | F´ φ80(*lac*Z)ΔM15 Δ(*lac*ZYA-*argF*) U169 *deoR* *recA*1 endA1 *hsdR*17(rₖ⁻ mₖ⁺) *phoA* *supE*44 λ⁻ thi-1 *gyrA*96 relA1. | From TransGen, China |
| *Escherichia*  *coli* BL21 | F⁻ *ompT hsdS*ₛ (rₛ⁻ mₛ⁻) *gal dcm*, λ(DE3). | From TransGen, China |
| *Escherichia*  *coli* 3064 | thrB1004 pro thi, *rpsL* *hsdS*, *lacZ*ΔM15 RP4-1360, Δ(*araBAD*)567, Δ*dapA*1341 :: [erm pir]. | From BioVector NTCC, China |

| **Table S8. Primers used in this study** | | | |
| --- | --- | --- | --- |
| Primers | Sequence | Description | |
| qPRecA-F | GTCTCAGGCATTGCGTAA | Quantitative PCR of gene *recA* | |
| qPRecA-R | AACATCACACCGATCTTCAT |  |  |
|  |  |  | |
| qPC-F | ATGACCATCAAGCGAATC | Quantitative PCR of gene *dddC* | |
| qPC-R | TGACTTCTTGGTGGCTAT |  |  |
|  |  |  | |
| qPA-F | GAAGATCGGACTGGAATA | Quantitative PCR of gene *dddA* | |
| qPA-R | AGATTAGGATGCTCATACT |  |  |
|  |  |  | |
| qPN-F | AAGCATTGTCCTGGATAT | Quantitative PCR of gene *acuN* | |
| qPN-R | GACTAGCACATCAGACTC |  |  |
|  |  |  | |
| qPK-F | GATGGCTGAGAAGTCCTT | Quantitative PCR of gene *acuK* | |
| qPK-R | CAATGATGGGTTTGGAGAT |  |  |
|  |  |  | |
| qPT-F | TATCTTGTCCGACATCAATG | Quantitative PCR of gene *dddT* | |
| qPT-R | GAACAGCGAATCACCTAC |  |  |
|  |  |  | |
| qPD-F | ATGTTCTATGTGGTCTGT | Quantitative PCR of gene *dddD* | |
| qPD-R | GTCATCGTAGATACCTAATAC |  |  |
|  |  |  | |
| qPI-F | GCAACGCTAAAAGACATC | Quantitative PCR of gene *acuI* | |
| qPI-R | CATCCTTGTAGTTGAGAGTA |  |  |
|  |  |  | |
| DT-F | GCGATTAAGCGTTGCATTGG | Detection of co-transcription among *genes* | |
| DT-R | GAACCCAAGCGATCCATTCG |  |  |
| TK-F | GCAGCATTACTCGAAGGCTC |  |  |
| TK-R | GGAGATTGGCTTGGTTGCTC |  |  |
| KN-F | GAGTGCATTCAGACTCTTCG |  |  |
| KN-R | GCTCTTTCGAGAAACGAGCC |  |  |
| NA-F | CCTTGTTCCTGTTGAAGCAG |  |  |
| NA-R | CTGGTGATATCGGTACCACC |  |  |
| AC-F | CCAGTGATAGTTGTCACGG |  |  |
| AC-R | GCATGGTTGGCATCAACGTG |  |  |
| ZH-F | CCTGCATGATCGACCTAGTG |  |  |
| ZH-R | CTCAGTTAGCACCTCTAGTCC |  |  |
| HI-F | CTTTCTGGATTGGCTGGGAA |  |  |
| HI-R | GTTGAGCAACACTGCGTCAC |  |  |
|  |  |  | |
| CZ-F | CGTCAGCTTGGGAAGCTAGC | Construction of Δ*Z* | |
| CZ-R | GACACGCTACCTTCATGAGC |  |  |
| T-F | GCGATTAAGCGTTGCATTGG |  |  |
| SG1-R | GAAGTGGCGCGTGGTATCGATATCGGTACCCCGGGGTTCAA |  |  |
| Zup-F | TTGAACCCGGGGTACCGATATCGATACCACGCGCCAC |  |  |
| Zup-R | CCGACATCCAATTCGCGGTGGAATGCCTCCTTACAAATG |  |  |
| Zdn-F | CATTTGTAAGGAGGCATTCCACCGCGAATTGGATGTCGG |  |  |
| Zdn-R | CCAGGGTTTTCCCAGTCACGACGCGGCCGCGTATGGCCCTCACAATGACC |  |  |
| Pq1-F | ctaAGCCGCGCGAATTCGAGCTCGGTATTGACAGCTAGCTCAGTCCTAGGTATAATACTAGTgTTTGAGCTTGAGGCTGACGGGTTTTAGAGCTAGAAATAG |  |  |
| knZ-F | GCTCGATTCGCTTGATGGTC |  |  |
| knZ-R | GTCTTGGCGTGACAATCCAG |  |  |
|  |  |  | |
| ComZ-F | GTTTTCCCAGTCACGACGCGGCCCTGCCTCTCTTCTTATGTTG | Construction of complementary plasmid pSE-*Z* | |
| ComZ-R | ATATTTAAAAGCAGCGGGAGCTTCACAATGACCAGATATCGGC |  |  |
|  |  |  | |
| acuZ -F | AAGAAGGAGATATACATATGCATGACCTTAAGGCGCTACG | Construction of plasmid pET-*acuZ* | |
| acuZ -R | TGGTGGTGGTGGTGCTCGAGCAATGACCAGATATCGGC |  | |
|  |  |  | |
| AcuZ-F | AAGAAGGAGATATACATATGACAACTACCCAACTCCCCAAG | Construction of plasmid pET-*acuR* | |
| AcuR-R | TGGTGGTGGTGGTGCTCGAGGCGGGGCAAGCCTTGAAG |  | |
|  |  |  | |
| 79Y-F | GCGAAGTAGGCGCCGGCGTGCTCTATCACGGC | Construction of AcuR mutants | |
| 79Y-R | GCCGTGATAGAGCACGCCGGCGCCTACTTCGC |  |  |
| 82Y-F | AGTTTGGCAGCGAAGGCGGCGCCGTAGTGCTC |  |  |
| 82Y-R | GAGCACTACGGCGCCGCCTTCGCTGCCAAACT |  |  |
| 83F-F | TCCAGTTTGGCAGCGGCGTAGGCGCCGTAGTG |  |  |
| 83F-F | CACTACGGCGCCTACGCCGCTGCCAAACTGGA |  |  |
| 86K-F | CAAGTGGCGATCCAGTGCGGCAGCGAAGTAGGCG |  |  |
| 86K-R | CGCCTACTTCGCTGCCGCACTGGATCGCCACTTG |  |  |
| 105F-F | AGGCATCTTCGACGGCGGTCTCCAGGCGCG |  |  |
| 105F-R | CGCGCCTGGAGACCGCCGTCGAAGATGCCT |  |  |
| 113F-F | TAACCATGGCGTGCCGCGCCAGCAGAGGCATC |  |  |
| 113F-R | GATGCCTCTGCTGGCGCGGCACGCCATGGTTA |  |  |
| 115R-F | CCGATAACCATGGGCTGCCATGCCAGCAGAG |  |  |
| 115R-R | CTCTGCTGGCATGGCAGCCCATGGTTATCGG |  |  |
| 116H-F | CCCGCCGATAACCAGCGCGTGCCATGCCAG |  |  |
| 116H-R | CTGGCATGGCACGCGCTGGTTATCGGCGGG |  |  |
| 120R-F | CCTACCAAGCAACCCGCCCGATAACCATGGCG |  |  |
| 120R-R | CGCCATGGTTATCGGGCGGGTTGCTTGGTAGG |  |  |
| 179F-F | CCTTCCCAGCCAATCCAGGCAGCTTTAGCTAAGAGGTC |  |  |
| 179F-R | GACCTCTTAGCTAAAGCTGCCTGGATTGGCTGGGAAGG |  |  |
| 180W-F | GCGCCTTCCCAGCCAATCGCGAAAGCTTTAGCTAAGAG |  |  |
| 180W-R | CTCTTAGCTAAAGCTTTCGCGATTGGCTGGGAAGGCGC |  |  |
| 183W-F | ATCACGGCGCCTTCCGCGCCAATCCAGAAAGC |  |  |
| 183W-R | GCTTTCTGGATTGGCGCGGAAGGCGCCGTGAT |  |  |
|  |  |  | |
| 129T-F | GCACCAAGCCACTGGCGCCATGATGTAAGTC | Construction of AcuZ mutants | |
| 129T-R | GACTTACATCATGGCGCCAGTGGCTTGGTGC |  |  |
| 130S-F | GAATCCAGCACCAAGCCAGCGGTGCCATGATGTAAGTC |  |  |
| 130S-R | GACTTACATCATGGCACCGCTGGCTTGGTGCTGGATTC |  |  |
| 174-F | CTTCAGGGGCAATGATGTTGGCAGCGAAATACGTCAGTTCAA |  |  |
| 174-R | TTGAACTGACGTATTTCGCTGCCAACATCATTGCCCCTGAAG |  |  |
| 199W-F | TGGCGTGACAATCGCGGGCAGGGCGGCC |  |  |
| 199W-R | GGCCGCCCTGCCCGCGATTGTCACGCCA |  |  |
| 201V-F | GCGTCTTGGCGTGGCAATCCAGGGCAG |  |  |
| 201V-R | CTGCCCTGGATTGCCACGCCAAGACGC |  |  |
| 202T-F | GAGCGTCTTGGCGCGACAATCCAGGGC |  |  |
| 202T-R | GCCCTGGATTGTCGCGCCAAGACGCTC |  |  |
| 206-F | CGGTAGTGAACGGCGCGTCTTGGCGTG |  |  |
| 206-R | CACGCCAAGACGCGCCGTTCACTACCG |  |  |
| 208-F | GCCAGTAACCGGTAGGCAACGGAGCGTCTTGG |  |  |
| 208-R | CCAAGACGCTCCGTTGCCTACCGGTTACTGGC |  |  |
| 231-F | GCAGGGCTCCGCATCGACCTGGGCCACACG |  |  |
| 231-R | CGTGTGGCCCAGGTCGATGCGGAGCCCTGC |  |  |
| 232E-F | CGATCATGCAGGGCGCCTGATCGACCTGG |  |  |
| 232E-R | CCAGGTCGATCAGGCGCCCTGCATGATCG |  |  |
| 235M-F | CGCACTAGGTCGATCGCGCAGGGCTCCTGATC |  |  |
| 235M-R | GATCAGGAGCCCTGCGCGATCGACCTAGTGCG |  |  |
| 247L-F | GGCATCACGGGCTGCGGCGAGTCCAACG |  |  |
| 247L-R | CGTTGGACTCGCCGCAGCCCGTGATGCC |  |  |
|  |  | |  |
| cF | AACACACAACAGGAGATCTGGCGTACGGAAAAATAACACGGCGAATACCCCATCACAATGACCAGATATCGG | | Construction of plasmid pEd47-C and pEd47-ZC |
| ZF | AACACACAACAGGAGATCTGGCGTACGGAAAAATAACACGGCGAATACCCCAGAATGCCTCCTTACAAATGATC | |  |
| dR | GGTGGTGGTGGTGCTCGAGTCACGTCGGGAGATACTCTC | |  |
|  |  |  | |
| iF | GCTCTAGAACTAGTGGATCCGGCGTACGGAAAAATAACACGGCGAATACCCGGGCCATACAGGACTAGGTG | Construction of plasmid pBd47-I and pBd47-RI | |
| rF | GCTCTAGAACTAGTGGATCGGCGTACGGAAAAATAACACGGCGAATACCCCTAAACGAGGCTTCTTCAGGC |  |  |
| iR | GTCGACGGTATCGATAAGCTTTAGGTCATCGTCACCACCAC |  | |

**References**

1. Qin Q, Ling C, Zhao Y, Yang T, Yin J, Guo Y, Chen GQ. CRISPR/Cas9 editing genome of extremophile *Halomonas* spp. *Metabolic Engineering.* (2018): 47, 219-229. <https://doi.org/10.1016/j.ymben.2018.03.018>.
2. Tang BL, Yang J, Chen XL, et al. A predator-prey interaction between a marine *Pseudoalteromonas* sp. and Gram-positive bacteria. *Nature Communications.* (2020): 11, 285. <https://doi:10.1038/s41467-019-14133-x>.
3. Kovach ME, Phillips RW, Elzer PH, Roop RM 2nd, Peterson KM. pBBR1MCS: a broad-host-range cloning vector. *Biotechniques*. (1994): 16, 800-802. <https://pubmed.ncbi.nlm.nih.gov/8068328/>.
